# New Insights Into the Biology of Protein O-GlcNAcylation: Approaches and Observations

**DOI:** 10.3389/fragi.2020.620382

**Published:** 2021-03-12

**Authors:** Toni Mueller, Xiaosen Ouyang, Michelle S. Johnson, Wei-Jun Qian, John C. Chatham, Victor Darley-Usmar, Jianhua Zhang

**Affiliations:** ^1^ Department of Pathology and Center for Free Radical Biology, University of Alabama at Birmingham, Birmingham, AL, United States; ^2^ Biological Sciences Division, Pacific Northwest National Laboratory, Richland, WA, United States

**Keywords:** OGT, OGA, Thiamet G, antibodies, click chemistry, in vivo

## Abstract

O-GlcNAcylation is a protein posttranslational modification that results in the addition of O-GlcNAc to Ser/Thr residues. Since its discovery in the 1980s, it has been shown to play an important role in a broad range of cellular functions by modifying nuclear, cytosolic, and mitochondrial proteins. The addition of O-GlcNAc is catalyzed by O-GlcNAc transferase (OGT), and its removal is catalyzed by O-GlcNAcase (OGA). Levels of protein O-GlcNAcylation change in response to nutrient availability and metabolic, oxidative, and proteotoxic stress. OGT and OGA levels, activity, and target engagement are also regulated. Together, this results in adaptive and, on occasions, detrimental responses that affect cellular function and survival, which impact a broad range of pathologies and aging. Over the past several decades, approaches and tools to aid the investigation of the regulation and consequences of protein O-GlcNAcylation have been developed and enhanced. This review is divided into two sections: 1) We will first focus on current standard and advanced technical approaches for assessing enzymatic activities of OGT and OGT, assessing the global and specific protein O-GlcNAcylation and 2) we will summarize *in vivo* findings of functional consequences of changing protein O-GlcNAcylation, using genetic and pharmacological approaches.

## Introduction

**FIGURE 1:**
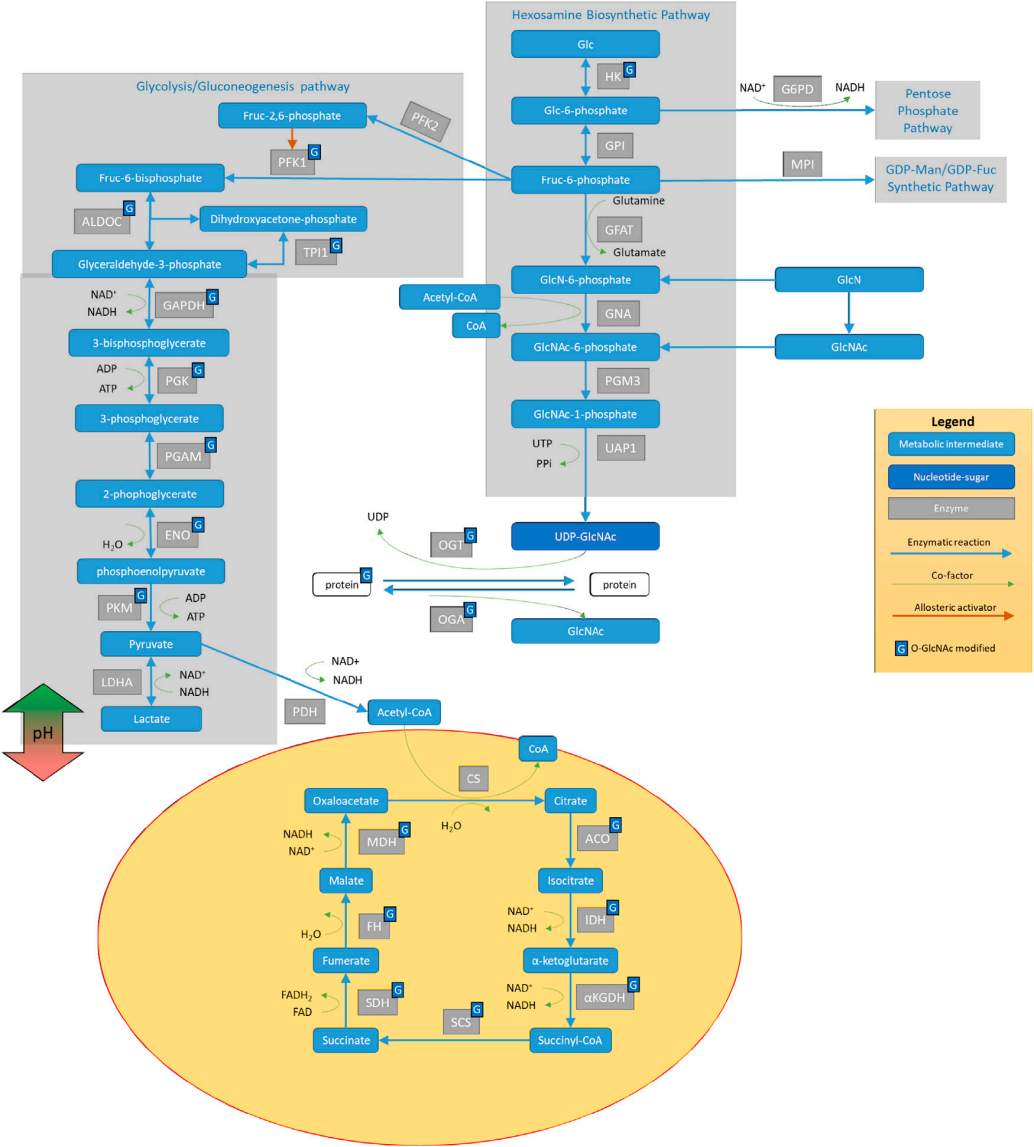
The interplay of the hexosamine biosynthetic pathway (HBP) with other glucose metabolic mechanisms. Glucose (Glc) metabolism proceeds through hexokinase (HK), which can then be diverted to the pentose phosphate pathway via the enzymatic activities of G6PD. Further down the pathway after GPI, Glc can proceed to 1) glycolysis (as diagramed on the left side) to provide pyruvate for the TCA cycle in the mitochondria, 2) the GDP-Man/GDP-Fuc synthetic pathway, or 3) the Hexosamine Biosynthetic Pathway (HBP). HBP metabolizes glucose (Glc) and converts it to UDP-GlcNAc with steps involving hexokinase (HK), glucose-6-phosphate isomerase (GPI), glutamine fructose-6-phosphate amidotransferase (GFAT), GlcN-6-P acetyltransferase (GNA), phosphoglucomutase 3 (PGM3), and UDP-N-acetylglucosamine pyrophosphorylase 1 (UAP1). UDP-GlcNAc serves as a substrate for protein modification on serine/threonine residues via the activities of O-GlcNAc transferase (OGT) that adds GlcNAc to proteins and O-GlcNAcase (OGA), which removes GlcNAc from proteins. GlcNAc can then re-enter HBP via N-acetylglucosamine kinase (NAGK) to generate N-acetylglucosamine 1-phosphate. Many of the enzymes involved in glucose metabolism and those involved in protein O-GlcNAcylation can be O-GlcNAcylated.

O-GlcNAcylation is a protein posttranslational modification highly sensitive to nutrient availability and stress, which modifies proteins associated with several cellular metabolic pathways (Figure 1). O-GlcNAcylation is controlled by the availability of its substrate UDP-GlcNAc and precursors, as well as the activities of O-GlcNAc transferase (OGT), which adds O-GlcNAc to proteins, and O-GlcNAcase (OGA), which removes O-GlcNAc from modified proteins (Hardiville and Hart, 2014; Bond and Hanover, 2015; and Wani et al., 2017a). It is known that this pathway is critical for controlling the relationship between aging and metabolism. In animals, the levels of global protein O-GlcNAcylation change with age (Fulop et al., 2008; and Liu et al., 2012). For example, in *C. elegans*, *oga-1* deletion increased and *ogt-1* deletion decreased worm lifespan (5). Interestingly, these lifespan changes are reversed under conditions of proteotoxic stress such as that induced by pathogenic tau, β-amyloid, and polyglutamine, known neurotoxic stressors linked to neurodegeneration in humans (6).

The importance of O-GlcNAcylation in maintaining normal cell function in mammalian organisms has been demonstrated by the three lines of evidence. 1. Mechanistically, protein O-GlcNAcylation, primarily regulated by OGT and OGA levels and activities, can regulate gene transcription and proteasomal activities, as well as autophagy (Wells et al., 2001; Zhang et al., 2003; Wani et al., 2015; Wani et al., 2017c; Dodson et al., 2018; and Zhang et al., 2018). 2. Genetically, OGT mutations are associated with X-linked intellectual disability in humans (Niranjan et al., 2015; Vaidyanathan et al., 2017; Willems et al., 2017; Selvan et al., 2018; Pravata et al., 2019; Pravata et al., 2020a; and Pravata et al., 2020b). In rodents, an OGA splice variant is linked to a genetic model of diabetes in the Goto-Kakizaki (GK) diabetic rat (Akimoto et al., 2007), while OGT and OGA gene disruption are either embryonic or perinatal lethal in mice (O'Donnell et al., 2004; Shafi et al., 2000; Watson et al., 2014; and Yang et al., 2012). 3. Pharmacological inhibitors have been developed, and the effects of inhibition of OGA have been assessed in different models of disease. The OGA inhibitor thiamet G is of special interest as it has an IC50 in the nM range and oral delivery of thiamet G decreases pathogenic tau phosphorylation in the brain in transgenic mice overexpressing tau (Yuzwa et al., 2008; Yuzwa et al., 2012). These and similar observations have resulted in an increased interest in O-GlcNAc biology in various cells and tissues in response to diverse signals.

Traditionally, the overall O-GlcNAcylation levels in an experimental system have been detected by western blot analyses using a few antibodies developed against a handful of O-GlcNAcylated proteins. A key assumption is that the levels of O-GlcNAcylation of these proteins represent the overall O-GlcNAcylation levels of most other cellular proteins (Arnold et al., 1996; Comer et al., 2001). This assumption is not without its caveats. For example, the antigens used for generating anti-O-GlcNAc antibodies are from abundant proteins in the cell. The epitopes these antibodies recognize do not represent all possible epitopes for cellular O-GlcNAcylated protein sites. Thus, the signals from antibody-based assays for total levels of O-GlcNAcylation can only give limited information on the levels of O-GlcNAcylation of less abundant proteins such as those involved in cell signaling. A less biased approach using chemoenzymatic tagging coupled with click chemistry has been developed for the investigation of protein O-GlcNAcylation (Thompson et al., 2018). Mass spectrometry-based strategies (Alfaro et al., 2012; Wang et al., 2014; Wang et al., 2017; and Thompson et al., 2018) are now increasingly being adopted to determine how specific protein O-GlcNAcylation is regulated. Recent studies have also begun to tackle the more challenging question of how the intracellular localization of specific O-GlcNAcylated proteins is changed in response to various physiological and pathological conditions.

To provide practical considerations of the various approaches to study the role and regulation of protein O-GlcNAcylation, in this review, we cover several major areas. For approaches, we discuss the following four aspects: a) The determination of the activities of the enzymes OGT and OGA. b) The assessment of overall and specific O-GlcNAcylation levels in cells/tissues using antibody or click chemistry methods. c) The determination and quantification of protein O-GlcNAc modification by mass spectrometry (MS). d) The determination of intracellular localization and function of O-GlcNAcylated proteins *in vitro* and *in vivo*. For studies in humans and animal models that shed light on biological function of protein O-GlcNAcylation, we discuss a) observations in humans, b) observations in genetically modified mouse models, and c) observations using potent pharmacological agents. We conclude with research questions and future directions in O-GlcNAc biology and its role in health and disease.

### Approaches for Assessing Enzymatic Activities of OGT and OGA and for Assessing the Global and Specific Protein O-GlcNAcylation

To understand the functions of protein O-GlcNAcylation, it is important to be able to measure the two enzymes that add and remove O-GlcNAc from proteins and the extent of overall protein O-GlcNAcylation in a given tissue or underdefined conditions. The following four sections provide an overview of these approaches. Furthermore, the development of methods to assess the location of specific O-GlcNAcylated proteins and the function of specific modifications *in vitro* and *in vivo* will also be highlighted.

#### Measurement of OGT and OGA Activity

OGT and OGA are the key enzymes for the addition and removal of O-GlcNAc modifications; thus, their levels and activity are important for sensing nutrient availability and metabolic, proteotoxic, and oxidative stress. These enzymes then modulate O-GlcNAcylation of target proteins to control the activities of proteins in response to changing nutrients, changing metabolites, and accumulations of toxic proteins and oxidants. Methods to assess the levels and localization of OGT and OGA proteins and other posttranslational modifications (e.g., phosphorylation) are now standard using western blots and immunocytochemistry and, thus, not included in this review. Instead, we focus on assessing OGT and OGA activities in cells and tissues (Table 1). Sources for key chemicals are also indicated where appropriate.

**TABLE 1:** OGT and OGA activity assays.

Assay	Substrate and measurement	Application
OGT activity	UDP-[^3^H]GlcNAc; CKII aa340-352; measure µCi GlcNAc incorporated (Zachara et al., 2011)	Can be performed with crude preparations (∼20–50 μg protein) or purified OGT enzyme (∼0.2–1.0 μg protein), sensitive to salt inhibition (Zachara et al., 2011; Groves et al., 2017)
OGT activity	Measure chemosensor binding, since it binds stronger with UDP than UDP-sugar (Kim, 2011)	The chemosensor binds more strongly to UDP than to the UDP-GlcNAc nucleotide-sugar donor and may be used to detect changes in O-GlcNAc incorporation. The chemosensor used may have nonspecific interactions with other cellular components
OGT activity	Measure ligand displacement using fluorescent UDP-GlcNAc analogs and an active sOGT enzyme (Kim, 2011)	Purified enzyme is needed. This method can be used for the screening of OGT inhibitors
OGA activity	Measure absorbance or fluorescence changes for synthetic substrates pNP-β-GlcNAc (400 nm) (Zachara et al., 2011) or 4MU-β-GlcNAc (excitation 360 nm, emission 450 nm) (Groves et al., 2017)	Can be performed with cell extracts (20–50 μg protein); GalNAc can be included in the reaction to inhibit lysosomal hexosaminidases A and B at a concentration that does not inhibit OGA, or the activities on GalNAc substrates can be subtracted from those on GlcNAc substrates (Zachara et al., 2011; Groves et al., 2017)

OGT enzymatic activity is best measured using standard biochemical assays. These assays quantify products in a time-dependent manner using appropriate substrates. The traditional method uses radioisotope-labeled UDP-[^3^H]GlcNAc and a known OGT target peptide CKII aa340-352. UDP-[^3^H]GlcNAc incorporated into the CKII peptide can be measured, and OGT activity can be calculated. Negative controls containing a mimic of the CKII peptide with Ser/Thr replaced with Ala and a negative control without a lysate need to be included. Nonradioactive OGT assays have also been developed using either a chemosensor or a ligand displacement method (Kim, 2011). The chemosensor method is based on the higher affinity of the sensor for binding UDP vs. UDP-GlcNAc; thus, a chemosensor-detected increase in UDP represents a measure of OGT activity. The ligand displacement method is based on fluorescent UDP-GlcNAc analogs, which have stronger fluorescence signals when binding to OGT. Neither of the nonradioactive methods matches the specificity and applicability of the radioactive assay (Kim, 2011). *In vitro* activity assays using recombinant OGT (Alteen et al., 2020) have been developed for screening OGT inhibitors, and these studies are not the focus of this review.

OGA activity can be determined using a fluorogenic substrate 4-methylumbelliferyl-*N*-acetyl-β-d-glucosaminide (4MU-GlcNAc) (**Sigma M9659**). 4-Methylumbelliferyl (4MU) has excitation and emission wavelengths of 360 and 450 nm, respectively. When conjugated to GlcNAc (4MU-GlcNAc), fluorescence is quenched. After removal of GlcNAc by OGA, 4MU fluorescence can be quantified and the results can be corrected for the background emission from buffer components and uncleaved 4MU-GlcNAc (**Sigma M2133**). To control for potential activity of lysosomal hexosaminidases, reactions using tissue homogenates or cell lysates should include a physiological excess of GalNAc, and parallel reactions for each sample using an exogenous GalNAc-substrate in place of the GlcNAc-substrate should be included on the same plate.

#### Detection of O-GlcNAcylated Proteins

OGT and OGA enzymatic activities are important. But, in many circumstances, their activities are simultaneously increased or decreased in response to biological stimuli. Therefore, the biological consequences of their regulation have to be further assessed by determining both the overall protein O-GlcNAcylation and O-GlcNAcylation of specific proteins. We first discuss antibody-based approaches that many laboratories, with the ability to do immunoprecipitation and western blots, can use. Then, we discuss a click chemistry-based derivatization method which can complement antibody-based approaches.

##### Detection of O-GlcNAcylated Proteins by Antibodies

There are several related methods to detect O-GlcNAcylated proteins; each method has unique characteristics according to the sensitivity and specificity of substrate detection. Due to the labile nature of the modification, it is important to minimize freeze/thawing of the samples and consider including OGA inhibitors in the sample preparations. The succinylated wheat germ agglutinin (sWGA) method is used to pulldown O-GlcNAc proteins to facilitate their identification (Monsigny et al., 1980; Zachara et al., 2011). While sWGA interacts with O-GlcNAcylated proteins, it lacks specificity. Routine inclusion of BSA-AP-GlcNAc, ovalbumin (positive controls), and BSA-AP (negative control) controls is recommended (Zachara et al., 2011).

The most widely used O-GlcNAc-specific antibodies are the monoclonal antibodies CTD110.6 and RL2 (Table 2). CTD110.6 was the first widely accepted O-GlcNAc antibody used in the field. It was raised against position 5 Ser modified RNA polymerase II subunit I C-Terminal Domain YSPTS*PS. CTD110.6 can recognize O-GlcNAc modifications of both Ser and Thr residues, verified *in vitro* using Ser-O-GlcNAc and Thr-O-GlcNAc, where Ser/Thr are not surrounded by any other amino acids (Comer et al., 2001). The binding of CTD110.6 to O-GlcNAcylated proteins can be competed with UDP-GlcNAc (Comer et al., 2001). This observation has been used to support its specificity (Comer et al., 2001). CTD110.6 can be used in immunoprecipitation. However, elution of glycoproteins from the antibody may be inefficient. Additionally, it is unclear how adjacent amino-acid sequences may provide charge or structural hindrance for antibody recognition. Subsequent investigations also revealed that, in addition to labeling O-GlcNAc, CTD110.6 also recognizes N-GlcNAc2-modified proteins (Isono, 2011) and terminal β-glycans on the complex N-glycans (Tashima and Stanley, 2014). To distinguish O-GlcNAc vs. N-GlcNAc2 modification of proteins, protein N-glycosidase F (PNGase F) can be used to remove N-linked glycans prior to CTD110.6 binding. Mild on-blot β-elimination is used to remove O-glycans based on the fact that the O-glycosidic bond is more labile than the N-glycosidic bond (Reeves et al., 2014). Blocking with 3% milk has also been shown to differentially decrease the affinity to N-GlcNAc2-modified proteins using nitrocellulose membranes, presumably because of the high concentration of glycoproteins and carbohydrates in milk (Reeves et al., 2014). The same study, however, showed that limited number of proteins can be precipitated by CTD110.6 (Reeves et al., 2014).

**TABLE 2:** Characteristics of the most commonly used O-GlcNAc antibodies.

	Antigen	Specificity
CTD110.6 (Cell Signaling mAb #9875)	O-GlcNAc modified peptide: YSPTS*PS	Can recognize O-GlcNAc-Ser, or O-GlcNAc-Thr. Cross reacts with N-GlcNAc2-modified proteins (Isono, 2011) and terminal β-glycans on complex N-glycans (Tashima and Stanley, 2014). Off-target binding may be alleviated by using PNGase F to remove N-linked glycans, and mild on-blot β-elimination was used to remove O-glycans
RL2 (Millipore MABS157)	Nuclear Envelope (NE) fractions from the rat liver	Adding galactose to the O-GlcNAc moiety or removing O-GlcNAc from the proteins decreased RL2 recognition of these proteins. It no longer recognizes NE antigen once they are partially proteolyzed. (Holt et al., 1987; Snow et al., 1987; Zachara et al., 2011; and Reeves et al., 2014)
1F5.D6(14), 9D1.E4(10), and 18B10.C7(3) (Millipore)	CKII peptide GSTPVS(β-O-GlcNAc)SANM	Bind BSA-CGSTPVS(β-*O*-GlcNAc)SANM not BSA-CGSTPVSSANM Inhibited by GSTPVS(β-*O*-GlcNAc)SANM, but not GSTPVSSANM or β-*O*-GlcNAc-Ser Binding to glycopeptide by 1F5.D6(14) (but not 9D1.E4(10) and 18B10.C7(3) only marginally) can also be inhibited by VS(β-*O*-GlcNAc)S and PVS(β-*O*-GlcNAc)SA

RL2 is another anti-O-GlcNAc antibody which was raised against rat liver nuclear envelope proteins and found to recognize 8 nuclear pore complex proteins, all of which are O-GlcNAc modified (Holt et al., 1987; Snow et al., 1987). Adding galactose to the O-GlcNAc moiety or removing O-GlcNAc from the proteins decreased RL2 recognition of these proteins, suggesting that the O-GlcNAc moiety contributes to the RL2 binding. However, RL2 cannot recognize partially digested forms of the 8 nuclear pore complex proteins, suggesting that, in addition to being O-GlcNAc modified, specific protein sequences and/or structures are also required for RL2 binding (Holt et al., 1987). Likely because of these structural constraints and in contrast to the CTD110.6 monoclonal antibody, the RL2 antibody does not recognize N-GlcNAc2-modified proteins (Reeves et al., 2014). A comparison of CTD110.6, RL2, and a few other additional antibodies has been described (Zachara et al., 2011; Reeves et al., 2014).

In addition to CTD110.6 and RL2, there are other O-GlcNAc antibodies produced using a 3-component immunogen with an O-GlcNAc-containing peptide derived from casein kinase II (CKII) α subunit (GSTPVS(β-O-GlcNAc)SANM), an MHC class II restricted helper T-cell epitope, and a TLR2 agonist as an adjuvant (Teo et al., 2010). 1F5.D6(14), 9D1.E4(10), and 18B10.C7(3) were found to selectively bind GSTPVS(β-*O*-GlcNAc)SANM and are now commercially available (Teo et al., 2010). These antibodies identified 132, 69, and 101 O-GlcNAcylated proteins in HEK293T cells cultured with an OGA inhibitor PUGNAc. In the same cell culture, 43 O-GlcNAcylated proteins were identified by CTD110.6, and a combined total of 215 proteins were identified with the four antibodies [CTD110.6, 1F5.D6(14), 9D1.E4(10), and 18B10.C7(3)] (Teo et al., 2010).

##### Detection of O-GlcNAcylated Proteins by Click Chemistry-Based Derivatization

**FIGURE 2:**
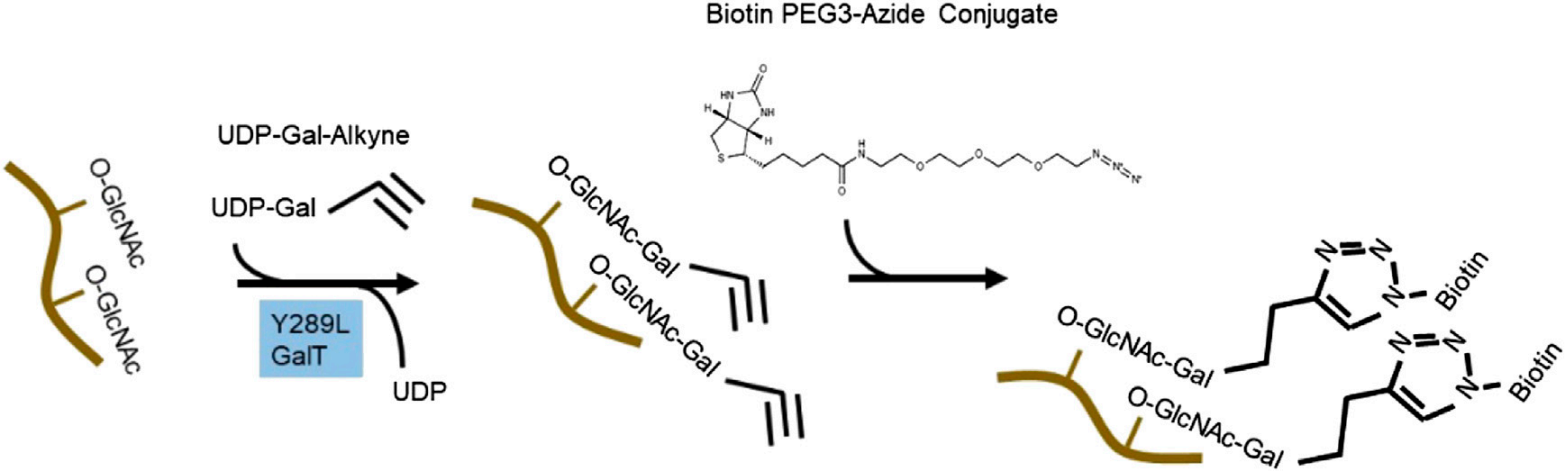
Click chemistry to enrich O-GlcNAcylated protein. An engineered bovine β-1,4-galactosyltransferase 1 (Y289L GalT) tags O-GlcNAcylated proteins with UDP-Gal-Alkyne. Through click chemistry, biotin is added to the modified proteins and can be used for pulldown and western blot analyses.

To circumvent the limitation of epitope preference for the antibodies, unbiased methods to enrich and detect O-GlcNAcylated proteins have been developed. One particular method of high sensitivity is to use chemoenzymatic labeling with click chemistry to enrich O-GlcNAcylated proteins for downstream identification by mass spectrometry or western blot analysis (Thompson et al., 2018). To do this, a recombinant bovine β-1,4-galactosyltransferase 1 (Y289L GalT) is used to tag O-GlcNAcylated proteins with an N-azidoacetylgalactosamine (GalNAz) group, with the Y289L mutation allowing the binding pocket high capacity for UDP-GalNAz (Figure 2). The azide handle can be used in the copper-catalyzed azide–alkyne cycloaddition (CuAAC) click reaction to attach biotin to O-GlcNAcylated proteins, allowing western blotting and streptavidin pulldown (**Thermofisher C33368, C33372**). Polyethylene glycol (PEG) tags of defined molecular mass provide the ability to reveal the stoichiometry of O-GlcNAcylation on proteins of interest (Thompson et al., 2018).

#### Identification and Quantification of Protein O-GlcNAcylation by Mass Spectrometry

**FIGURE 3:**
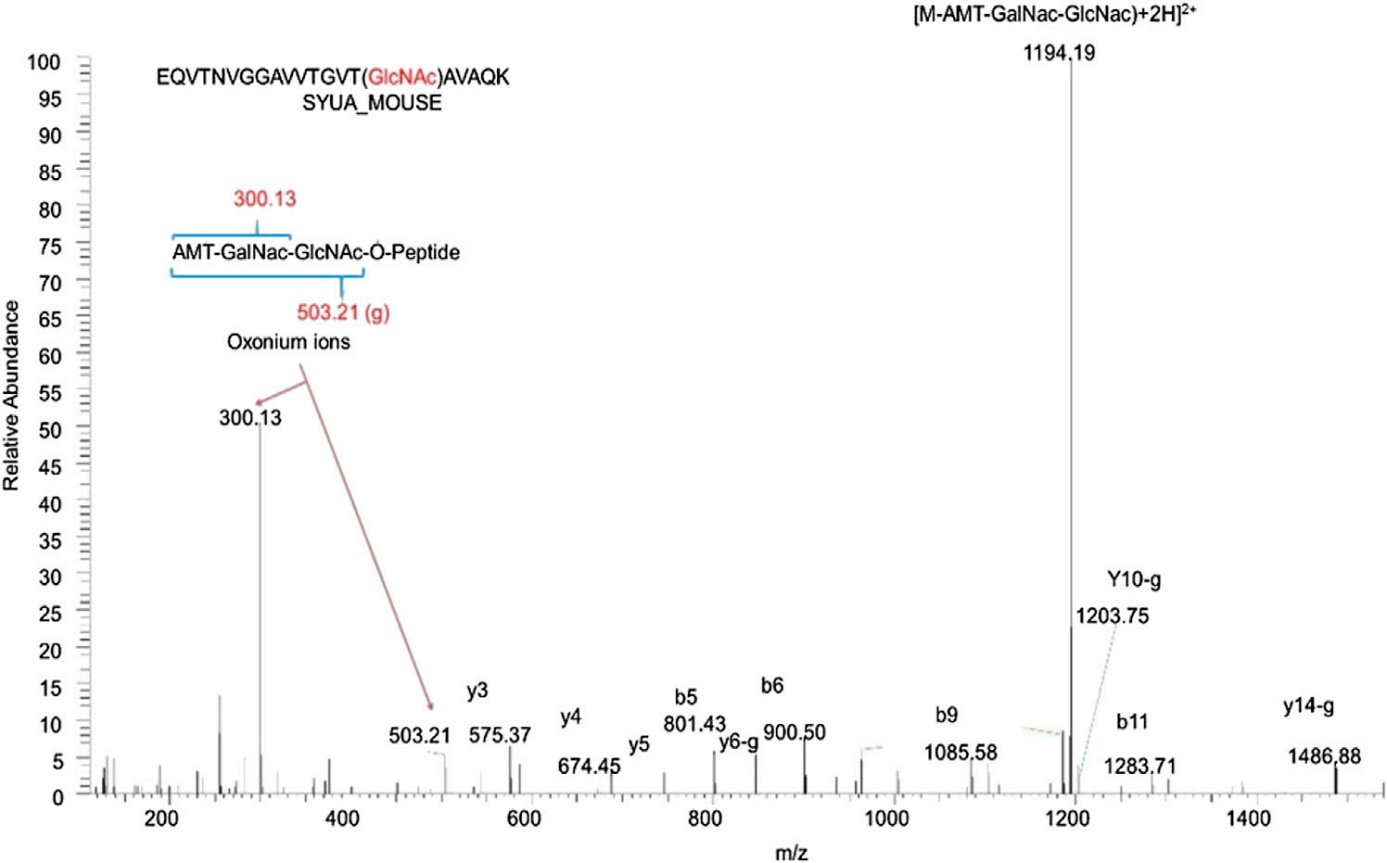
Identification of α-synuclein as an O-GlcNAcylated protein in the mouse brain by mass spectrometry. 10 mg wet hippocampal tissues were processed and digested by trypsin. Protein digests containing O-GlcNAc peptides were identified by the chemical/enzymatic photochemical cleavage method in which O-GlcNAc was tagged with the aminomethyltriazolacetylgalactosamine (AMT-GalNac). The labeled O-GlcNAc peptides were identified with the high-energy collisional dissociation (HCD) method. The letter “g” indicates the neutral loss of the entire AMT-GalNac-GlcNac moiety. This is a representative peptide identified as α-synuclein amino acid 58–77 with T75 O-GlcNAcylated.

Mass spectrometry- (MS-) based approaches have been reported as promising strategies for identification of specific amino-acid residues of O-GlcNAcylation and quantification of the levels of modification. Antibody- and lectin-based O-GlcNAc enrichment strategies followed by MS analyses have been reported (Nagel et al., 2013; Wang et al., 2007). However, this strategy has been challenging due to the lack of specificity of the affinity reagents. Alternatively, the same chemoenzymatic click chemistry method can be used with Y289L GalT to tag O-GlcNAcylated proteins with a GalNAz group, followed by the copper-catalyzed azide–alkyne cycloaddition (CuAAC) click reaction to attach a cleavable biotin to O-GlcNAcylated proteins for MS analyses (Figure 2). Then, the tagged O-GlcNAc peptides are released via photocleavage by UV, followed by LC-MS/MS analyses (Chalkley et al., 2009; Ma and Hart, 2017; Riley and Coon, 2018). Due to the labile nature of the modification, traditional collision-induced dissociation (CID) may break the O-linkage between sugar modification and Ser/Thr. New fragmentation approaches are now used to identify the site of O-GlcNAcylation. HCD (higher-energy collision dissociation) (Ma and Hart, 2017) and ETD (electron transfer dissociation) (Chalkley et al., 2009; Ma and Hart, 2017; Riley and Coon, 2018) have been applied for O-GlcNAc site identification. In a typical study, protein lysates can be trypsin digested and multiplex labeled using tandem mass tags and then subjected to enrichment and HCD-LC-MS/MS. An example of such a study is illustrated in Figure 3. HCD will detect both the peptide carrying the modification and the O-GlcNAc containing oxonium ions (Wang et al., 2017). Recent studies also demonstrated that native mass spectrometry (Leney et al., 2017) without tagging may also be a useful alternative tool for quantitative analysis of O-GlcNAcylation. However, high-resolution mass spectrometry will be required for such detection since the mass shift of O-GlcNAcylation is relatively small compared to large proteins and complexes (+203Da).

#### Determination of Intracellular Localization and Function of O-GlcNAcylated Proteins *In Vitro* and *In Vivo*

Once we know that a protein is O-GlcNAcylated, the next question may be where we find the modified protein and whether the O-GlcNAcylated protein has a different function than the unmodified. Because of the lack of antibodies recognizing site-specific O-GlcNAc modifications and the lack of suitable amino-acid mimetics to replace the Ser/Thr, these tasks are much more difficult than the equivalent studies with protein phosphorylation. We discuss here a few approaches that may help address these questions.

##### Intracellular Imaging Technique to Label O-GlcNAc

For many proteins, their intracellular locations are also critical to serve their biological function. It is, therefore, important to be able to determine whether modification of proteins by O-GlcNAc changes their intracellular location or, conversely, whether protein intracellular locations affect their O-GlcNAc modification. In addition to subcellular fractionation methods (not discussed in this review), one technique that enables detection of overall protein O-GlcNAcylation involves labeling cells with the UDP-azidogalactose substrate (UDP-GalNAz) and Y289L GalT, followed by biotin-alkyne, and then probing with a streptavidin-AlexaFluor 488 conjugate (Clark et al., 2008). This method can help determine whether there is an increase or decrease in O-GlcNAcylation in specific cellular compartments.

FRET is another technique used to detect the location of specific O-GlcNAcylated proteins. A known protein can be tagged with EGFP, and then, cells expressing the EGFP-protein can be treated with Ac4GalNAz which is intracellularly metabolized to UDP-GalNAz and attached to O-GlcNAc modified proteins. Alkyne-TAMRA can be conjugated to the azide. When EGFP and TAMRA are in close proximity, a FRET donor-acceptor pair is formed. The change of fluorescence indicates that this specific protein is O-GlcNAcylated (Lin et al., 2015; Doll et al., 2016; Hu et al., 2018). To ensure this works specifically, negative controls without EGFP, Ac4GalNAz, or the alkyne-TAMRA need to be included. The development of a one-step metabolic feeding strategy with a fluorescent glucosamine-nitrobenzoxadiazole (GlcN-NBD), which serves as a metabolic precursor for protein O-GlcNAcylation, can also enable the identification of O-GlcNAcylated proteins in live cells (Tan et al., 2018).

##### Functional Studies of Proteins With Specific O-GlcNAc Modification *In Vitro*

*In vitro* biochemical methods can be used to determine the consequence of site-specific O-GlcNAcylation on protein structure, as well as its effect when applied to cultured cells. One such approach involves chemically synthesizing a short peptide with only one Ser or Thr residue and adding O-GlcNAc to the residue via incubation with OGT and UDP-O-GlcNAc. The short peptide can be ligated to the N-terminal and C-terminal of the protein (synthesized in *E.coli* using bacterial expression vectors) using expressed protein ligation (EPL). This strategy has been effectively used to generate semisynthetic site-specific O-GlcNAcylated α-synuclein, which is relatively small and does not natively contain Cys residues. Because EPL relies on the interaction of N-terminal cysteine (Cys) and a C-terminal thioester group on the peptides to be ligated, a Cys residue can be introduced in the recombinant expressed C-terminal peptide of α-synuclein and the O-GlcNAcylated peptide can be synthesized with an N-terminal thioproline and C-terminal thioester group. Following the first ligation step attaching the O-GlcNAcylated peptide to the C-terminal fragment, thioproline becomes the most N-terminal residue of the assembled peptide. The thioproline is then chemically transformed to produce an N-terminal Cys. This intermediate peptide can then be ligated to the N-terminal peptide, which is expressed with a C-terminal thioester group. Following assembly into a full-length protein, non-native Cys residues can be converted back to native alanine (Ala) residues by radical desulfurization (Marotta et al., 2015; Lewis et al., 2017; Levine et al., 2019). The number and position of native Cys and/or Ala residues present in a target protein can present some challenges in utilization of this synthetic process, and the native sequence of the O-GlcNAcylated protein must be considered.

##### Functional Studies of Proteins With Specific O-GlcNAc Modification *In Vivo*

Many proteins have multiple sites of O-GlcNAcylation; therefore, mutagenesis methods have been used to determine whether changing one or more sites of O-GlcNAcylation affects protein function (Rexach et al., 2012). The mutated proteins can be reintroduced to cells either through exogenous expression via the transduction or transfection method or through CRISPR/Cas editing. Recent techniques have also aided substrate-/site-specific O-GlcNAcylation by nanobody (a single-domain peptide or protein-specific antibody which is of small size ∼12 kDa)-OGT (Ramirez et al., 2020). Engineered nanobody-OGT can selectively target tagged proteins by proximity through nanobody recognition of its target protein, while global elevation of O-GlcNAcylation can be suppressed by truncation of the tetratricopeptide repeat domain in OGT that recognizes global OGT targets. The validity of the approach has been demonstrated to be effective for selectively increasing α-synuclein O-GlcNAcylation in HEK293T cells using the nanobody that recognizes the EPEA sequence on α-synuclein (Ramirez et al., 2020). The same nanobody-OGT can be used on EPEA-tagged JunB or Nup62 and, presumably, other proteins if individually tagged (Ramirez et al., 2020).

Studies of protein O-GlcNAcylation function may require identification of proteins that only interact with the O-GlcNAcylated glycoform of the protein. Crosslinking methods have been optimized for this purpose (Yu et al., 2012a; Pham et al., 2013; Wu and Kohler, 2019). This requires stable transfection of cells with a mutant (F383G) of the UDP-GlcNAc pyrophosphorylase (UAP1) and feeding cells with synthesized GlcNDAz-1-P which has a diazirine photocrosslinker appended to GlcNAc. GlcNDAz can be attached to Ser or Thr by endogenous OGT, and upon UV irradiation, O-GlcNAcylated proteins can be crosslinked with their interacting proteins. This method has been demonstrated in HeLa cells with proteins that interact with O-GlcNAc-modified nucleoporins and may aid studies of O-GlcNAc biology (Yu et al., 2012a; Pham et al., 2013; Wu and Kohler, 2019).

Although there are multiple traditional and advanced approaches to assess the functional relevance of specific protein O-GlcNAcylation, one must be cautious regarding the interpretation of observations as any of the approaches with mutations and tags of either OGT or the protein targets may change their intracellular function. Method validation and specificity need to be carefully examined.

### *In Vivo* Findings of Functional Consequences of Changing Protein O-GlcNAcylation, Using Genetic and Pharmacological Approaches

To further understand the functional consequence of changing protein O-GlcNAcylation, in the following three sections, we discuss a) studies of human disease, b) investigations using genetically modified mouse models with elevated or suppressed overall protein O-GlcNAcylation, and c) the effects of pharmacological inhibitors of O-GlcNAcase (OGA).

#### Investigation of the Function of O-GlcNAcylated Proteins in Human Samples

The importance of O-GlcNAcylation has been demonstrated in human diseases primarily based on two approaches. Genetic studies have linked OGT mutations to X-linked intellectual disability in humans (Niranjan et al., 2015; Vaidyanathan et al., 2017; Willems et al., 2017; Selvan et al., 2018; Pravata et al., 2019; Pravata et al., 2020a; and Pravata et al., 2020b). Alternatively, specimens from many human diseases have been analyzed for global and specific protein O-GlcNAcylation and for OGT and OGA levels and activity.

X-linked intellectual disability (XLID) exhibits abnormalities both in the brain and in the body, including the lips, ears, eyes, genitals, and fingers (Pravata et al., 2020b). Earlier studies using X chromosome exome sequencing in samples from patients with X-linked intellectual disability (XLID) have identified potentially pathogenic variants of OGT and a few other genes (Niranjan et al., 2015). Further studies identified a missense substitution of L254F in the 7th tetratricopeptide repeat (TPR) domain of OGT, which was segregated with XLID (Vaidyanathan et al., 2017). Affected lymphoblastoids exhibited decreased OGT protein levels and half-lives, resulting in altered gene expression transcriptomes, decreased OGA levels, and apparently unchanged levels of global O-GlcNAcylated protein as measured by western blot analyses using CTD110.6 antibodies (Vaidyanathan et al., 2017). An independent study at the same time identified both an R284P missense mutation and a splicing defect in the TPR domain of OGT (Willems et al., 2017). Similarly, these resulted in decreased OGT and OGA levels in patient-derived fibroblasts and unchanged global protein O-GlcNAcylation as assessed by the RL2 antibody (Willems et al., 2017). Subsequently, other OGT mutations (A259T and E339G in the TPR domain) have also been identified that segregate with XLID in affected families. Knock-in of these mutants using CRISPR/Cas9 into a male human embryonic stem cell line did not alter global protein O-GlcNAcylation as measured by western blot analyses using CTD110.6 antibodies, but did change gene expression profiles (Selvan et al., 2018). These studies suggest that the overall protein O-GlcNAcylation levels can be maintained by compensatory downregulation of OGA when OGT activities are decreased. OGT mutation may also change its nonenzymatic activities including by changing its location or its interaction with other cellular molecules. Change of location or level of specific protein O-GlcNAcylation target may be another possible mechanism by which pathology is induced.

A catalytic domain N567K mutation in OGT has also been identified in XLID (Pravata et al., 2019). Editing this mutation into *Drosophila* resulted in decreased global protein O-GlcNAcylation in head lysates using the RL2 antibody (Pravata et al., 2019). Nonetheless, editing the mutation into human male embryonic stem cells did not change global protein O-GlcNAcylation as measured by western blot analyses using the RL2 antibody, presumably due to concurrently decreased OGA levels in the edited cells (Pravata et al., 2019). Despite the lack of a global change in O-GlcNAcylation, differentiation and processing of Host Cell Factor 1 (HCF1) are perturbed (Pravata et al., 2019). These studies highlighted the importance of OGT in cellular function and that western blot analyses using CTD110.6 or RL2 antibodies, which fail to identify global alterations of O-GlcNAcylation, are insufficient in the determination of substrate-specific abnormalities related to altered OGT function. As more studies emerge, a new finding demonstrated that N648Y mutation in the catalytic domain of OGT, indeed, caused decreased global protein O-GlcNAcylation that are evident from western blot analyses using RL2 antibody, in edited human embryonic stem cells (Pravata et al., 2020a), indicating that some OGT defects persist and are not corrected by cellular compensatory mechanisms. Table 3 summarizes OGT mutations that are associated with XLID.

**TABLE 3:** OGT mutations associated with XLID and the impact on cellular function.

Mutation	Cells	OGT and OGA levels	Global protein O-GlcNAcylation as measured by western blot analyses	Other cellular functions	References
L254F in the TPR domain	Affected lymphoblastoids	↓ OGT protein levels and half-life, ↓OGA levels	Unchanged (CTD110.6 antibody)	Altered gene expression transcriptomes	Vaidyanathan et al. (2017)
R284P and a splicing defect in the TPR domain	Patient-derived fibroblasts	↓ OGT and OGA protein levels	Unchanged (RL2 antibody)		Willems et al. (2017)
A259T and E339G in the TPR domain	Edited human embryonic stem cell line		Unchanged (CTD110.6 antibody)	Changed gene expression profiles	Selvan et al. (2018)
N567K in the catalytic domain	Edited human embryonic stem cells	↓ OGA protein levels	Unchanged (RL2 antibody)	Perturbed differentiation and processing of Host Cell factor 1	Pravata et al. (2019)
N567K in the catalytic domain	Edited Drosophila		↓ in head lysates (RL2 antibody)		
N648Y mutation in the catalytic domain	Edited human embryonic stem cells		↓ (RL2 antibody)		Pravata et al. (2020a)

In addition to information provided in Table 3, which summarizes specific mutations demonstrated to segregate with XLID, it has also been reported that changes in OGT and OGA levels and altered global protein O-GlcNAcylation are associated with human diseases (Chatham et al., 2020). For example, in Alzheimer’s disease, global protein O-GlcNAcylation was shown to be increased in detergent insoluble fractions using ELISA and HGAC85/39 antibodies (Griffith and Schmitz, 1995) and using western blot analyses with the CTD110.6 antibody, as well as decreased levels and activities of OGA protein while OGT protein levels remain unaffected (Förster et al., 2014). However, decreased global protein O-GlcNAcylation was also reported in postmortem Alzheimer’s disease brains compared to controls as measured by radioimmuno-dot-blot analyses with an RL2 antibody (Liu et al., 2004), as well as western blots with RL2 (Pinho et al., 2019). Using western blot analyses with the CTD110.6 antibody, we also found increased overall protein O-GlcNAcylation levels in Parkinson’s disease stage IV patients (Wani et al., 2017b). In hippocampal and cortical samples from human temporal lobe epilepsy (TLE) patients, global protein O-GlcNAcylation has been found to be decreased compared to controls, as measured by western blot using CTD110.6 antibody (Sanchez et al., 2019). These studies suggest that both increased and decreased protein O-GlcNAcylation can be detrimental to health. Additionally, one major concern with these antibody-based studies is that observations are biased by epitope preference and the relative abundance of specific proteins (also see Table 2). The development of state-of-the-art quantitative proteomics will certainly provide more information regarding the landscape of the protein O-GlcNAcome in control and disease-carrying specimens. One such study has found 12 peptides to exhibit decreased O-GlcNAcylation and 119 peptides to exhibit increased O-GlcNAcylation in Alzheimer’s disease brains compared to controls, providing more precision and specificity for a better understanding of protein O-GlcNAcylation in diseases (Wang et al., 2017).

In addition to the brain, where levels of OGT, OGA, and O-GlcNAcylated proteins are high, other tissues may also exhibit altered O-GlcNAcylation in human diseases. For example, increases in protein O-GlcNAcylation, OGT, and OGA were found in cardiac biopsies from patients with aortic stenosis compared to samples from nonhypertrophied hearts (Lunde et al., 2012). In apical core tissues removed during LVAD implantation in heart failure patients, there was a ∼20% increase of overall protein O-GlcNAcylation levels as observed using a click chemistry method, compared to patients without heart failure (Dassanayaka et al., 2017). In whole blood samples, decreased OGT and OGA mRNA levels were found in diabetic patients compared to controls (Coomer and Essop, 2014). In freshly isolated endothelial cells from forearm vein biopsy, overall protein O-GlcNAcylation levels were found increased as measured by immunofluorescence using the RL2 antibody, in patients with type 2 diabetes (T2DM) compared to nondiabetic controls (Masaki et al., 2020). Human skeletal muscle samples (vastus lateralis muscle before and after a 4 h euglycemic hyperinsulinemic clamp) from T2DM patients also exhibited elevated total protein O-GlcNAcylation before and after clamp, as measured by western blot analyses using the RL2 antibody (Shi et al., 2018).

In patients with alcoholic liver cirrhosis, global protein O-GlcNAcylation was shown to be decreased by western blot analyses with the RL2 antibody (Zhang et al., 2019). In patients with ulcerative colitis and Crohn’s disease, colon specimen immunostaining with RL2 revealed that both OGT and global protein O-GlcNAcylation are decreased in these inflammatory bowel diseases (Zhao et al., 2018). Studies also have reported increased protein O-GlcNAcylation in cancer cells, with worse outcomes associated with higher O-GlcNAc levels. For example, in lung and prostate cancers, higher OGT and O-GlcNAc levels have been demonstrated using RL2 immunohistochemistry compared to adjacent tissues (Mi et al., 2011). Poorer prognosis was associated with higher global protein O-GlcNAcylation in prostate cancers as measured by immunohistochemistry using CTD110.6 antibody (Kamigaito et al., 2014). Colon tumors have also been shown to exhibit higher OGT and protein O-GlcNAcylation as assessed by western blots with the RL2 antibody (Olivier-Van Stichelen et al., 2014).

These observations are consistent with the concept that O-GlcNAc regulation is important for normal cellular function in humans (Table 4). Both in tissues normally expressing high levels of OGT and OGA and in tissues with low levels of these enzymes, changes of overall protein O-GlcNAcylation, OGT, and OGA have been observed in pathological samples.

**TABLE 4:** Altered levels of protein O-GlcNAcylation, OGT, and OGA levels, as well as OGA activity in human disease samples.

Specimen	OGT and OGA levels and activities	Protein O-GlcNAcylation	Ref
AD postmortem brains		↑ protein O-GlcNAcylation in detergent insoluble fractions observed using ELISA and HGAC85/39 antibodies	Griffith and Schmitz (1995)
AD postmortem brains	↓ OGA protein levels and activities, unchanged OGT levels	↑ protein O-GlcNAcylation observed using western blot analyses with the CTD110.6 antibody	Förster et al. (2014)
AD postmortem brains		↓ protein O-GlcNAcylation observed using radioimmuno-dot-blot analyses with RL2 antibody	Liu et al. (2004)
AD postmortem brains		↓ protein O-GlcNAcylation observed using western blots with RL2	Pinho et al. (2019)
AD postmortem brains		↓ O-GlcNAcylation of 12 peptides, and ↑ O-GlcNAcylation of 119 peptides, using quantitative proteomics	Wang et al. (2017)
PD postmortem brains		↑ protein O-GlcNAcylation levels in PD stage IV patients using western blot with the CTD110.6 antibody	Wani et al. (2017b)
TLE patient hippocampal and cortical samples		↓ protein O-GlcNAcylation observed by western blot using CTD110.6 antibody	Sanchez et al. (2019)
Myocardial biopsies from heart failure patients	↑ ncOGT, mOGT, sOGT, and ncOGA protein ↑ OGT mRNA	↑ protein O-GlcNAcylation levels by western blot with CTD110.6 antibody (no statistics since *n* = 3 each), in aortic stenosis left ventricular tissues, compared to patients with nonischemic areas with coronary artery disease	Lunde et al. (2012)
Apical cores removed prior to LVAD implantation		↑ protein O-GlcNAcylation levels by ∼20% as observed by click chemistry from apical cores removed during LVAD implantation in heart failure patients compared to patients without heart failure	Dassanayaka et al. (2017)
Diabetes whole blood samples	↓ OGT and OGA mRNA		Coomer and Essop (2014)
Endothelial cells in patients with T2DM		↑ protein O-GlcNAcylation levels by immunofluorescence with RL2, from freshly isolated endothelial cells from forearm vein J-wire biopsy from patients with T2DM compared to non-diabetic controls	Masaki et al. (2020)
Skeletal vastus lateralis muscle biopsy		↑ OGT in diabetic patients by western blot analyses with RL2	Shi et al. (2018)
Liver biopsy	↓ OGT and ↑ OGA protein levels	↓ protein O-GlcNAcylation levels from patients with alcoholic liver cirrhosis by western blots with the RL2 antibody	Zhang et al. (2019)
Colon tissues of IBD patients	↓ OGT	↓ protein O-GlcNAcylation levels in ulcerative colitis and Crohn’s disease patients using immunohistochemistry with RL2	Zhao et al. (2018)
Human lung and prostate cancers	↑OGT	↑ protein O-GlcNAcylation levels in lung and prostate cancers using immunohistochemistry with RL2 antibodies compared to adjacent tissues	Mi et al. (2011)
Human prostate cancers		↑ protein O-GlcNAcylation levels in prostate cancers with poorer prognosis as measured by immunohistochemistry using the CTD110.6 antibody	Kamigaito et al. (2014)
Human colon tumors	↑OGT	↑ protein O-GlcNAcylation levels in colon tumors vs. controls as measured with western blots with RL2	Olivier-Van Stichelen et al. (2014)

#### Investigation of the Function of O-GlcNAcylated Proteins in Mouse Models With Altered *Ogt*, *Oga*, or *Gfat* Gene Expression

Human samples provide evidence of disruptions of the O-GlcNAcylation pathway in pathological tissues. To determine whether perturbation of the pathway has any functional consequences, rodents have been used to investigate the relevance of overexpression, disruption, and inhibition of OGA and OGT. We discuss 1) genetically engineered mouse studies with decreased global protein O-GlcNAcylation due to *Ogt* deletions, focusing on development, cardiomyocyte, skeletal muscle, liver, adipose tissue, pancreatic beta cell, and selective neuronal populations, 2) mouse models with increased global protein O-GlcNAcylation, including exogenous expression of a dominant negative OGA, *Oga* knockout, and OGT and GFAT overexpression, and 3) mouse models with *Ogt* and *Oga* manipulation using viral delivery.

##### Ogt Deletions Affecting Mouse Development, Cardiomyocyte, Skeletal Muscle, Liver, Adipose Tissue, Pancreatic Beta Cells, and Neuronal Functions

Table 5 summarizes various *Ogt* deletion models and the reported findings demonstrating the critical roles of OGT in development and health. The *Ogt* gene is on the X chromosome in the mouse genome; thus, a single copy of the *Ogt* gene is present in the male genome. It has been demonstrated that *Ogt* gene deletion results in loss of embryonic stem cell viability (Shafi et al., 2000). Experimental *Ogt* deletion in rodent models can be achieved using a Cre-loxP recombination system, which deletes the *Ogt* gene in specific tissues and cell types and/or until induction of deletion by taking advantage of specific ligand-induced activation or repression of Cre expression. This strategy has been used to generate several cell-type-specific mouse models. ZP3 (zona pellucida glycoprotein 3)-Cre mediated disruption of *Ogt* (deleting amino acid 206-232 of the 1037 amino-acid sequence of OGT) resulted in embryonic lethality (Shafi et al., 2000). Tamoxifen-inducible global *Ogt* knockout mice were generated by crossing *Ogt* floxed mice with R26-Cre-ERT2 (Ida et al., 2017). These mice exhibited lethality 4 weeks after tamoxifen injection, consistent with the finding that OGT is essential for organismal survival in adult mice (Ida et al., 2017). Knockout of *Ogt* in the placenta produced by crossing *Ogt* floxed mice with CYP19 (aromatase cytochrome P450)-Cre mice resulted in key features of the early prenatal stress phenotype. Adult offspring had decreased body weights and elevated corticosterone in response to restraint stress. Expression of genes related to mitochondrial function is perturbed, and cytochrome c oxidase activities are decreased in the hypothalamus of these knockout mice (Howerton and Bale, 2014).

**TABLE 5:** Mouse models with *Ogt* deletion. Most of the observed phenotypes are tissue dysfunctions, with a few exceptions of potential benefits in metabolism-related phenotypes (highlighted in bold).

*Ogt* deletion	Mouse model	Phenotype	Ref
Embryonic *Ogt* deletion	Crossing *Ogt* floxed mice with ZP3-Cre	Embryonic lethality	Shafi et al. (2000)
Tamoxifen-inducible global *Ogt* knockout	Crossing *Ogt* floxed mice with R26-Cre-ERT2	Lethality 4 weeks after tamoxifen injection	Ida et al. (2017)
Placenta *Ogt* deletion	Crossing *Ogt* floxed mice with CYP19-Cre	The adult offsprings had decreased body weights and elevated corticosterone in response to restraint stress. Expression of genes related to mitochondrial function are perturbed, and cytochrome C oxidase activities are decreased in the hypothalamus of the knockout mice	Howerton and Bale (2014)
Cardiomyocyte- *Ogt* deletion	Crossing *Ogt* floxed mice with α-MHC-Cre	Only 12% of mice survived to weaning age. Surviving mice exhibit ↓ body weight, pulmonary edema, diminished heart function, and signs of heart failure	Watson et al. (2014)
Early fetal cardiomyocyte-*Ogt* deletion	Crossing *Ogt* floxed mice with TnT-Cre mice	Heart developmental defects and neonatal lethality	Mu et al. (2020)
Tamoxifen inducible, cardiac *Ogt* deficient	Crossing *Ogt* floxed mice with αMHC-driven mutated estrogen receptor flanked cre (Mer-Cre-Mer, MCM)	↓ of *Ogt* mRNA and ↓ of global protein O-GlcNAcylation measured by western blot and immunofluorescence analyses using RL2 antibody Four weeks after tamoxifen, no cardiac dysfunction was observed in the unstressed heart ↑ infarction 4 weeks after tamoxifen and 24 h after permanent coronary ligation ↑ heart dysfunction and signs of heart failure 4 weeks after tamoxifen and 4 weeks after permanent coronary ligation infarction, ↓ protein O-GlcNAcylation as measured by Click chemistry	Watson et al. (2010)
		↑ cardiac dysfunction but not hypertrophy at 2 and 4 weeks after transverse aortic constriction (TAC) ↓ protein O-GlcNAcylation levels in the heart by ∼40 and 20%, respectively, at these time points, as measured by western blot using the RL2 antibody ↓ OGA mRNA transcript and protein levels at 2 weeks but not 4 weeks	Dassanayaka et al. (2017)
		Deleting *Ogt* starting 18 days after TAC surgery (with established ↑ LV posterior wall thickness, ↑ heart rate) caused ↓ of protein O-GlcNAcylation measured by western blot with RL2, ↑ left ventricular dysfunction, and ↑ cardiomyocyte cross-sectional area	Zhu et al. (2019)
**Skeletal muscle *Ogt* deletion**	**Crossing *Ogt* floxed mice with Mlc1f-Cre mice**	**↓ blood glucose during exercise; ↓ high-fat-diet-induced obesity and insulin resistance**	Murata et al. (2018)
**Skeletal muscle *Ogt* deletion**	**Crossing *Ogt* floxed mice with HSA-cre or HSA-rtTA/TRE-cre mice**	**↑ whole body energy expenditure and insulin sensitivity**	Shi et al. (2018)
Liver, skeletal muscle, adipose tissue, or pancreatic β-cell *Ogt* deletion	Crossing *Ogt* floxed mice with Alb-Cre, Mlc1f-Cre, Adipoq-Cre, and Pdx1PB-CreER	Knockout *Ogt* in the liver, skeletal muscle, or adipose tissue did not alter glucose metabolism, whereas knockout *Ogt* in pancreatic beta cells resulted in transient hypoglycemia, higher insulin secretion, adiposity, and subsequent hyperglycemia	Ida et al. (2017)
**Adipose tissue- *Ogt* deletion**	**Crossing *Ogt* floxed mice with Adipoq-Cre**	**↓ hyperphagia induced obesity; ↓ insulin resistance in response to HFD**	Li et al. (2018)
**Inducible adipose tissue- *Ogt* deletion**	**Crossing *Ogt* floxed mice with Adipoq-CreER**	**A rapid visceral fat loss**	Yang et al. (2020a)
Liver *Ogt* deletion	Crossing *Ogt* floxed mice with Alb-Cre	Hepatomegaly, fibrosis, inflammation, and necroptosis	Zhang et al. (2019)
Macrophage *Ogt* deletion	Crossing *Ogt* floxed mice with LyzM-Cre	Metabolic and inflammatory phenotypes noted by breeding *Ogt* floxed mice with vs. without LyzM-cre	Li et al., (2019), Yang et al. (2020b)
		↑ proinflammatory responses to bacterial endotoxin LPS in macrophages	Hasanain Al-Mukh et al. (2020)
T-cell *Ogt* deletion	Crossing *Ogt* floxed mice with lck-cre	T-cell apoptosis	O'Donnell et al. (2004)
Treg cell *Ogt* deletion	Crossing *Ogt* floxed mice with Foxp3^YFP-Cre^ mice	Progressive systemic autoimmune lesions and lethality at 4 weeks of age ↓ Foxp3, ↑ CD4^+^ and CD8^+^ T cells, ↑ effector/memory cell ratio with the CD4^+^ and CD8^+^ compartments in both the lymph nodes and the spleen, ↑ B cell frequency in the lymph nodes, ↑ IgG, IgM, and free kappa and lambda chains in the serum, and ↑T-helper Th1, Th2, and Th17 cells were prominent in these mice	Liu et al. (2019)
Intestinal epithelial *Ogt* deletion	Crossing *Ogt* floxed mice with Villin-cre	Gut inflammation	Zhao et al. (2018)
Intestinal epithelial *Ogt* deletion	Crossing *Ogt* floxed mice with Villin-cre	↓ body weight; change of gut microbiome	Zhao et al. (2020)
Neuron-specific *Ogt* deletion	Crossing *Ogt* floxed mice with Syn1-Cre	↓ frequency of pups born, ↓ sizes at birth, and ↑ phosphorylation of tau at postnatal day 9	O'Donnell et al. (2004)
Forebrain neuron *Ogt* deletion	Crossing *Ogt* floxed mice with CaMKIIα-Cre mice	↓ Body weight gain starting from postnatal week 7 neurodegeneration starting from 2 months of age	Wang et al. (2016)
Sensory neuron *Ogt* deletion	Crossing *Ogt* floxed mice with Nav1.8-Cre	↓ body weight, improved glucose tolerance, ↓ epidermal innervation, and deficits in sensory behavior	Su and Schwarz (2017)
Inducible sensory neuron *Ogt* deletion	Crossing *Ogt* floxed mice with brn3a-CreERT2	Adult neurodegeneration first observed in the nerve fibers, later at the cell body	Su and Schwarz (2017)
**Hypothalamus arcuate nucleus *Ogt* deletion**	**Crossing *Ogt* floxed mice with AgRP-Cre mice**	**↑ white adipose tissue thermogenic program, improves glucose tolerance, ↓ body weight gain, and improves insulin tolerance in response to a high-fat diet**	Ruan et al. (2014)
Inducible forebrain neuron *Ogt* deletion	Crossing *Ogt* floxed mice with CaMKIIα-CreERT mice	↑ adipose tissue, ↑ food intake, ↑ energy expenditure, ↑ activity, and ↑ obesity within 4 weeks of tamoxifen injection	Lagerlof et al. (2016)
		↑ food intake and ↑ obesity the initial peripheral insulin resistance after *Ogt* deletion was reversed after 2–4 months, and hypothalamus neuronal loss was evident	Dai et al. (2018)
		↓ learning and memory performance in RAWM and fear conditioning tests ↓ hippocampal synaptic spine density and proteins including NR1A, NR2B, PSD-95, and synapsin-1, while ↑ Schaffer collateral LTP	Wheatley et al. (2019)

Cardiomyocyte-specific deletion of *Ogt* has been generated by crossing *Ogt* floxed mice with an α-myosin heavy chain- (αMHC) driven Cre recombinase transgenic line. This cross resulted in postnatal lethality with only 12% of mice surviving to weaning age. Those that survived exhibit lower body weight, pulmonary edema, diminished heart function, and signs of heart failure (Watson et al., 2014). Early fetal cardiomyocyte-specific *Ogt* knockout generated by breeding *Ogt* floxed with TnT (cardiac Troponin T)-Cre mice also resulted in heart developmental defects and neonatal lethality (Mu et al., 2020). Tamoxifen-inducible, cardiac-specific *Ogt* deficient mice have been generated by crossing homozygous *Ogt* floxed mice with mice carrying αMHC-driven mutated estrogen-receptor-flanked Cre recombinase (Mer-Cre-Mer, MCM). After being injected with tamoxifen for 5 days, followed by “washout” for 5 days, cardiomyocyte-specific *Ogt* deletion was achieved, as demonstrated by ∼60% decrease of *Ogt* mRNA and ∼60% decrease of global protein O-GlcNAcylation measured by western blot and immunofluorescence analyses using the RL2 antibody (Watson et al., 2010). Furthermore, 4 weeks after tamoxifen injection, inducible cardiac-specific deletion of *Ogt* did not cause cardiac dysfunction in the unstressed heart, but exacerbated infarction and heart failure when mice were subjected to permanent coronary ligation (Watson et al., 2010). In a pressure overload model, inducible *Ogt* deletion in adult cardiomyocytes resulted in a decline in heart function 2 and 4 weeks after transverse aortic constriction (TAC) (Dassanayaka et al., 2017). Global protein O-GlcNAcylation levels from the heart were down by ∼40 and ∼20%, respectively, at these time points, as measured by western blot analyses using the RL2 antibody (Dassanayaka et al., 2017). A recent study also compared the impact of deleting *Ogt* before surgery vs. 18 days after TAC surgery on left ventricular function (Zhu et al., 2019). In this study, both pre- and postsurgery *Ogt* deletion resulted in decreased global protein O-GlcNAcylation as assessed by western blot analyses with the RL2 antibody. Deleting *Ogt* starting 18 days after TAC surgery with established pathological hypertrophy also exacerbated left ventricular dysfunction. There were changes noted in phospholamban and cardiac troponin phosphorylation, as well as PKA O-GlcNAcylation, although whether all these changes contributed to the pathology in this model needs further investigation.

Skeletal-muscle-specific deletion of *Ogt* has been generated by crossing homozygous *Ogt* floxed mice with skeletal-muscle-specific Mlc1f (myosin light chain 1)-Cre mice. This skeletal-muscle-specific *Ogt* deletion decreased blood glucose during exercise, decreased body mass, and alleviated insulin resistance in response to high-fat diet (Murata et al., 2018). HSA (human alpha-skeletal actin)-Cre-mediated muscle-specific *Ogt* knockout mice also exhibited increased whole body energy expenditure and insulin sensitivity (Shi et al., 2018). These findings suggest that, in contrast to effects on cardiomyocytes, decreasing OGT in skeletal muscles may be beneficial for energy metabolism and protect against T2DM.

Using Alb (albumin)-Cre, Mlc1f (myosin light chain 1)-Cre, Adipoq (adiponectin)-Cre, and Pdx1PB (pancreatic and duodenal homeobox 1 enhancer Pst-Bst fragment)-CreER™, liver, skeletal muscle, adipose tissue, and pancreatic beta cell-specific *Ogt* knockout mice were generated (Ida et al., 2017). Knockout of *Ogt* in the liver, skeletal muscle, or adipose tissue did not alter glucose metabolism, whereas knockout *Ogt* in pancreatic beta cells resulted in transient hypoglycemia, higher insulin secretion, adiposity, and subsequent hyperglycemia (Ida et al., 2017). A recent study with adipocyte deletion of *Ogt* using Adipoq-Cre has demonstrated a decrease in hyperphagia-induced obesity and a decrease in insulin resistance in response to high-fat diet (Li et al., 2018). In addition, mice with an inducible adipose tissue-specific *Ogt* knockout exhibited a rapid visceral fat loss (Yang et al., 2020a). Further analyses of liver-specific *Ogt* knockout mice identified phenotypes including hepatomegaly, fibrosis, inflammation, and necroptosis (Zhang et al., 2019). *Ogt* floxed mice with LyzM-Cre exhibited metabolic and inflammatory phenotypes compared to *Ogt* floxed mice without LyzM-Cre (Li et al., 2019; Yang et al., 2020b). Macrophages from these mice exhibit increased proinflammatory responses to bacterial endotoxin LPS (Hasanain Al-Mukh et al., 2020). T-cell-specific deletion of *Ogt* accomplished by breeding *Ogt* floxed mice with lck-Cre resulted in T-cell apoptosis (O'Donnell et al., 2004). Another recent study breeding *Ogt* floxed mice with Foxp3^YFP-Cre^ mice found that *Ogt* deletion in regulatory T cells resulted in progressive systemic autoimmune lesions and lethality at 4 weeks of age (Liu et al., 2019). Decreased Foxp3, increased CD4^+^ and CD8^+^ T cells, increased effector/memory cell ratio within the CD4^+^ and CD8^+^ compartments in both the lymph nodes and the spleen, upregulated B-cell frequency in the lymph nodes, and increased IgG, IgM, and free immunoglobulin kappa and lambda chains in the serum, as well as increased T-helper Th1, Th2, and Th17 cells, were prominent in these mice (Liu et al., 2019). Intestinal epithelial *Ogt* deletion by crossing *Ogt* floxed mice with Villin-Cre mice was first reported to increase the risk of gut inflammation (Zhao et al., 2018) and then also found to change the expression of gut hormone genes, decrease body weight, enhance glucose clearance rate, and alter gut microbiome (Zhao et al., 2020). Crossing *Ogt* floxed mice with Neurog3-Cre deleted *Ogt* in ngn3+ progenitors, did not change body weight, but resulted in higher initial blood glucose levels and faster glucose clearance before weaning (Zhao et al., 2020).

Neuron-specific *Ogt* mutagenesis using the Syn1-Cre transgene resulted in pups born at lower-than-expected frequency, much smaller sizes at birth, and increased phosphorylation of tau at postnatal day 9 (O'Donnell et al., 2004). Crossing *Ogt* floxed mice with CaMKIIα-Cre led to attenuated body weight gain starting from postnatal week 7 and neurodegeneration starting from 2 months of age (Wang et al., 2016). Crossing *Ogt* floxed mice with Nav1.8-Cre mice (which express Cre in sensory neurons in the dorsal root ganglia and trigeminal ganglia) resulted in decreased body weight, improved glucose tolerance, decreased epidermal innervation, and deficits in sensory behavior (Su and Schwarz, 2017). Crossing *Ogt* floxed mice with brn3a-CreERT2 mice (which express inducible Cre in dorsal root ganglia neurons) also resulted in adult neurodegeneration first observed in the nerve fibers and later at the cell body (Su and Schwarz, 2017). It was reported that crossing *Ogt* floxed mice with an AgRP (Agouti-related protein)-Cre promotes white adipose tissue thermogenic programming, improves glucose tolerance, decreases body weight gain, and improves insulin tolerance in response to a high-fat diet (Ruan et al., 2014). In contrast, crossing *Ogt* floxed mice with tamoxifen-inducible CaMKIIα-CreERT2 leads to increased adipose tissue, increased food intake, and development of obesity despite increased energy expenditure and activity, within 4 weeks of tamoxifen injection (Lagerlof et al., 2016). This *Ogt*-mediated hyperphagia is associated with feeding circuitry function in the paraventricular nucleus (PVN) (Lagerlof et al., 2016). The increase in food intake and body weight has been confirmed with an independent study (Dai et al., 2018), which also demonstrated that the initial peripheral insulin resistance after *Ogt* deletion was reversed after 2–4 months, and hypothalamic neuron loss was evident (Dai et al., 2018). Additionally, inducible *Ogt* deletion using CaMKIIα-CreERT2 resulted in learning and memory deficits as assessed by radial arm water maze (RAWM) and fear conditioning tests, while long-term potentiation (LTP) was increased in CA3-CA1 Schaffer collateral projection, hippocampal synaptic spine density was decreased, and levels of proteins including NR1A, NR2B, PSD-95, and synapsin-1 were decreased (Wheatley et al., 2019).

These studies of OGT function in diverse tissues support the conclusion that OGT is essential to many cellular functions. There are a few exceptions. For example, it has been shown that gene disruption of *Ogt* in skeletal muscles, adipose tissues, or AgRP neurons, using a loxP-Cre approach, improves metabolic phenotypes (Ruan et al., 2014; Li et al., 2018; Murata et al., 2018; Shi et al., 2018; and Yang et al., 2020a). Despite the identification of several OGT target proteins whose O-GlcNAcylation is decreased in response to the OGT knockout, we know little about how this alters their intracellular location, interactions with other cellular components, and other biological functions. Furthermore, whether the identified target proteins affected by OGT deletion are the key proteins contributing to a specific pathology is unclear.

##### Mouse Models With Increased Global Protein O-GlcNAcylation, Including With Exogenous Expression of a Dominant Negative OGA, Oga Knockout, and OGT and GFAT Overexpression

It is clear that deficient O-GlcNAcylation is associated with developmental disorders and dysfunction of many tissues as evident by the phenotypes of mice with *Ogt* knockout in diverse tissues and cells as summarized above. The functional consequences of excessive O-GlcNAcylation have also been investigated *in vivo* in mice with a) exogenous expression of a dominant negative OGA, b) *Oga* knockout, and c) OGT and GFAT overexpression. Table 6 summarizes phenotypes observed using these models. OGA is an enzyme which can both remove O-GlcNAc from Ser/Thr residues of proteins (as an O-GlcNAcase) and add an acetyl group to histone protein in an *in vitro* assay (Toleman et al., 2004). NCOAT^GK^ (*dnOga*) is an O-GlcNAcase-inactive splice variant of the *Oga* gene, which has been shown to function in a dominant-negative capacity to increase global protein O-GlcNAcylation levels when overexpressed in MCF10AT cells as measured by FL2 immunofluorescence (Bowe et al., 2006). Inducible expression of *dnOga* in various tissues has identified OGA function in these tissues *in vivo*.

**TABLE 6:** Mouse models with increased global protein O-GlcNAcylation, including with exogenous expression of a dominant negative OGA, *Oga* knockout, OGT, and GFAT overexpression. Most of the observed phenotypes are tissue dysfunctions, with a few exceptions of potential benefits in metabolism-related phenotypes (highlighted in bold).

Oga deletion	Mouse model	Phenotype	References
Inducible expression of dnOga in the mammary tissue	Crossing TRE-EGFP-NCOAT^GK^ mice with MMTV-rtTA	↑ protein O-GlcNAcylation as measured by immunohistochemistry using RL2, ↓ mammary ductal side-branching morphogenesis	Bowe et al. (2006)
		Blocked estrogen cell signaling	Whisenhunt et al. (2006)
Inducible expression of dnOga in the skeletal muscle	Crossing TRE-EGFP-NCOAT^GK^ mice with MCK-rtTA	↑ protein O-GlcNAcylation as measured by western blots using RL2, muscle atrophy, impaired mobility, and 70–80% morbidity in male mice 2–4 weeks after Dox	Huang et al. (2011)
Inducible expression of dnOga in the lens fiber cells	Crossing TRE-EGFP-NCOAT^GK^ mice with gamma-F-crystallin-rtTA	↑ Protein O-GlcNAcylation as measured by western blots using RL2, ↓ proteasome activity, ↑ in cataract surface area in the lenses, and inhibition of lens fiber cell denucleation (Wang et al., 2009)	Wang et al. (2009)
Embryonic Oga deletion	Insertion of the gene trap in the first intron	↑ protein O-GlcNAcylation in 20-month-old tissues compared to 4 months, as wells as *Oga* deletion cells and animals as measured by western blots using the CTD110.6 antibody MEFs exhibited mitotic defects, embryonic developmental delay, and perinatal lethality	Yang et al. (2012)
		**↑protein O-GlcNAcylation as measured by western blots using the CTD110.6 antibody** ***Oga*+/- are lean with ↓ fat mass, ↑ energy expenditure, and improved glucose tolerance and resistant to HFD-induced deficits in glucose metabolism, obesity, and hepatic steatosis**	Yang et al. (2015)
Ubiquitous Oga deletion (exon1 and promoter) starting at oocytes	Crossing Oga floxed mice with MMTV-Cre	3% of KO mice survived at weaning and exhibited ↓ *Oga* mRNA and protein, ↑ global protein O-GlcNAcylation as measured by western blots with RL2 KO mice exhibited hypoglycemia and low liver glycogen stores *Oga*+/- also exhibited altered metabolism as assessed using CLAMS. Only female *Oga*+/- mice exhibited ↑ weight gain compared to wildtype in response to HFD	Keembiyehetty et al. (2015)
Oga deletion (exon1 and promoter) in the nervous system	Crossing Oga floxed mice with Nestin-Cre	↑ protein O-GlcNAcylation as measured by western blots with RL2 KO mice exhibited brain development delay with hypopituitarism, early-onset obesity, and metabolic dysregulation	Olivier-Van Stichelen et al. (2017)
		PET analyses demonstrated that brain uptake of 18F-LSN3316612 (a high-affinity ligand of OGA) was reduced by 82% compared with control	Paul et al. (2019)
Inducible expression of shRNA of *Oga*	Doxycycline promoter-Oga-shRNA at Rosa26 locus, Dox for 10 days	↓ OGA mRNA was on average of 70–80%, ↓ binding to ^3^H-Thiamet G in brain homogenates of ∼80% ↑ 1.4 × protein O-GlcNAcylation in brain homogenates as assessed by a quantitative sandwich immunoassay using both wheat germ agglutinin and the RL2 antibody The decrease of OGA did not result in overt phenotypes	Hastings et al. (2017)
MHC-OGT; MHC-OGA		↑OGT in the heart results in adverse cardiac remodeling and premature death ↑OGA in the heart led to resistance to pathological stress induced by pressure overload	Umapathi et al. (2020)
GFAT↑ in skeletal muscle and fat	GLUT4 promoter	Insulin resistance	Hebert et al. (1996), McClain (2002), McClain et al. (2000)
GFAT↑ in liver	PEPCK promoter	Obesity, hyperlipidemia, impaired glucose tolerance, and insulin resistance	McClain, (2002), Veerababu et al., 2000)
GFAT↑ in β cells	RIP promoter	Hyperinsulinemia, obesity, and diabetes phenotypes	McClain, (2002), Tang et al. (2000)
Inducible Gfat1↑ or **Gfat1↓ in cardiomyocytes**	TRE-*Gfat1*:: αMHC-tTA; **αMHC-MCM::*Gfat1* floxed**	↑ Gfat1→ ↑ hypertrophic response to pressure overload; ↑ overall cardiac protein O-GlcNAcylation and mTOR activity. Inhibiting mTOR by rapamycin or inhibiting OGT by alloxan attenuated Gfat1 overexpression phenotype **↓ Gfat1→ ↓ hypertrophic response to pressure overload, ↓ overall cardiac protein O-GlcNAcylation, and mTOR activity**	Tran et al. (2020)

Doxycycline-inducible expression of *dnOga* in the mammary epithelium by crossing TRE-NCOAT^GK^ mice with MMTV (murine mammary tumor virus)-rtTA mice resulted in an increase of global protein O-GlcNAcylation as measured by immunohistochemistry with the RL2 antibody, decreased mammary ductal side-branching morphogenesis during pubertal development (Bowe et al., 2006), and blocked estrogen cell signaling (Whisenhunt et al., 2006). Doxycycline-inducible expression of *dnOga* in skeletal muscle by crossing TRE-NCOAT^GK^ mice with MCK-rtTA mice resulted in muscle atrophy, impaired mobility, with 70–80% morbidity in male mice 2–4 weeks after induction with doxycycline (Huang et al., 2011). Doxycycline-inducible expression of *dnOga* in lens fiber cells by crossing TRE-NCOAT^GK^ mice with gamma-F-crystallin-rtTA mice resulted in decreased proteasome activity, a ∼ 3-fold increase in cataract surface area in the lenses, and inhibition of lens fiber cell denucleation (Wang et al., 2009).

*Oga*-deficient mice were first generated by insertion of the gene trap vector in the first intron of *Oga*. *Oga* knockout embryonic fibroblasts exhibited mitotic defects and genomic instability. *Oga*-deficient offspring exhibited embryonic developmental delay and perinatal lethality (Yang et al., 2012). This study also demonstrated increased global protein O-GlcNAcylation in the 20-month-old mouse brain, lung, skin, and thymus using western blots with CTD110.6 antibody, as well as decreased OGT and OGA proteins, compared to tissues from an younger adult, suggesting perturbation of the O-GlcNAc pathway in aging (Yang et al., 2012). *Oga* heterozygotes are lean with decreased fat mass, increased energy expenditure, improved glucose tolerance, and resistance to high-fat-diet-induced deficits in glucose metabolism, obesity, and hepatic steatosis (Yang et al., 2015).

Mice carrying neofloxed *Oga* exon 1 and its promoter led to embryonic lethality (Keembiyehetty et al., 2015). Crossing *Oga* floxed mice with MMTV-Cre led to perinatal lethality. Only 3% of knockout mice survived at weaning and exhibited loss of *Oga* mRNA and protein and an increase of global protein O-GlcNAcylation as measured by western blots with the RL2 antibody (Keembiyehetty et al., 2015). Knockout mice also exhibit hypoglycemia and low liver glycogen stores (Keembiyehetty et al., 2015). Heterozygous mice with exon 1 and promoter deletion showed altered metabolism as assessed using the Comprehensive Lab Animal Monitoring System (CLAMS). Only female heterozygous mice exhibited increased weight gain compared to wildtype in response to high-fat diet (Keembiyehetty et al., 2015).

*Oga* deletion in the nervous system by crossing mice with *Oga* exon 1 and its promoter floxed with Nestin-Cre resulted in an increase of global protein O-GlcNAcylation in the brain as assessed by western blot using the RL2 antibody (Olivier-Van Stichelen et al., 2017). PET analyses demonstrated that brain uptake of 18F-LSN3316612 (a high-affinity ligand of OGA) was reduced by 82% compared with control (Paul et al., 2019). These knockout mice exhibited brain development delay with hypopituitarism, early-onset obesity, and metabolic dysregulation (Olivier-Van Stichelen et al., 2017).

*Oga* downregulation has also been achieved using an inducible expression of shRNA over the whole body (Hastings et al., 2017). After doxycycline for 10 days, OGA mRNA was down on average of 70–80%, and binding to ^3^H-Thiamet G in brain homogenates was down ∼80%. The decrease of OGA did not result in overt phenotypes (Hastings et al., 2017). The mice were normal with a 1.4 × increase of protein O-GlcNAcylation in brain homogenates as assessed by a quantitative sandwich immunoassay using both wheat germ agglutinin and the RL2 antibody (Hastings et al., 2017).

Since it has been shown that OGT deficiency leads to decreased overall protein O-GlcNAcylation, it is not surprising that OGA deficiency leads to an increase of overall protein O-GlcNAcylation. Similar to the studies of OGT function, OGA deficiency also causes pathologies in almost all of the tissues investigated. The only exception is that the *Oga* heterozygous mice seem to have improved whole body metabolism (Yang et al., 2015). Whether increased specific protein O-GlcNAcylation due to OGA deficiency is responsible for the observed pathologies is still unclear.

OGT overexpression in the heart has been demonstrated to result in adverse cardiac remodeling and premature death (Umapathi et al., 2020), while OGA overexpression in the heart led to resistance to pathological stress induced by pressure overload (Umapathi et al., 2020). This is the second example, in addition to the aforementioned *Oga* heterozygotes, that changing the OGA level might be beneficial in certain pathological conditions. Overexpression of GFAT (glutamine:fructose-6-phosphate amidotransferase), an important enzyme in the HBP pathway, in skeletal muscle and fat tissue in transgenic mice using the GLUT4 (Glucose transporter type 4) promoter, results in increased leptin and insulin resistance (Hebert et al., 1996; McClain et al., 2000; McClain, 2002). The model is phenotypically identical to several animal models of glucose toxicity and diabetes, as well as to human T2DM. GFAT overexpression in the liver of transgenic mice using the PEPCK (phosphoenolpyruvate carboxykinase) promoter eventually leads to obesity and insulin resistance (Veerababu et al., 2000; McClain, 2002). GFAT overexpression in β cells in transgenic mice using the RIP (rat insulin II) promoter also led to hyperinsulinemia, obesity, and diabetes phenotypes (Tang et al., 2000; McClain, 2002). A recent study used TRE-Gfat1 and αMHC-tTA to induce Gfat1 expression in cardiomyocytes after 2 weeks of doxycycline withdrawal. While basal heart function, histology, and the expression of unfolded protein response and other hexosamine biosynthetic pathway genes appear normal, an exacerbated hypertrophic response to pressure overload was observed. In contrast, in mice with tamoxifen-inducible cardiomyocyte-specific knockout of Gfat1, an attenuated hypertrophic response to pressure overload was observed (Tran et al., 2020). In this study, pressure overload increased overall cardiac protein O-GlcNAcylation and mTOR activity, and Gfat1 overexpression resulted in further mTOR upregulation. Rapamycin or OGT inhibitor alloxan attenuated the Gfat1 overexpression phenotype. In contrast, Gfat1 knockout decreased overall cardiac protein O-GlcNAcylation and mTOR activation. These studies suggest that some of the detrimental phenotypes of over-O-GlcNAcylation may be associated with mTOR activation (Tran et al., 2020). Taken together, increasing GFAT has been shown to be detrimental in multiple tissues, and decreasing GFAT may be beneficial in the heart in resisting pressure-overload-induced hypertrophy via mTOR inhibition.

##### Mouse Models With Ogt Manipulation Using Viral Delivery

Aside from the use of Cre-LoxP-mediated recombination, viral delivery of *Ogt* has been used to investigate the effect of OGT overexpression *in vivo* (Table 7). Bilateral lentiviral delivery of *Ogt* into the hippocampus improved contextual fear conditioning test performance in young mice and improved both radial arm water maze (RAWM) and contextual fear conditioning test performance in aged mice (Wheatley et al., 2019). Systemic delivery of WT, tagged, or mutated *Ogt* using adenovirus is another method to evaluate effects of OGT overexpression *in vivo* (Yang et al., 2008). Using this method, *Ogt* overexpression in the liver was found to result in insulin resistance (Yang et al., 2008), advance the phase of *Bmal1* and *Clock* gene expression, and increase the levels of *Per2*, *Cry1*, *Rorγ*, and *Dbp* during the peak phase (Li et al., 2013). Decrease of liver *Ogt* by injecting *Ogt* floxed mice with adenoviral Cre resulted in poorer glucose tolerance at ZT1, advanced circulating glucose by 6–8 h, decreased *Bmal1* amplitude, and decreased O-GlcNAcylation of BMAL1 and CLOCK proteins (Li et al., 2013). Conversely, OGT knockdown in the liver using adenovirus with OGT shRNA decreased gluconeogenesis, ameliorated diabetes, and improved glucose homeostasis in db/db mice (Ruan et al., 2012). Studies have also shown that rAAV6-OGT led to maladaptive cardiac remodeling and fibrosis and that rAAV6-OGA attenuated LV remodeling in diabetic mice (Prakoso et al., 2018).

**TABLE 7:** Mouse models with *Ogt* manipulation using viral delivery. Potential benefits highlighted in bold.

*Ogt* change	Mouse model	Phenotype	Ref
**OGT ↑ in the brain**	**Bilateral ventricle lentiviral *Ogt***	**↑ Contextual fear conditioning test performance in young mice, ↑ Both RAWM and contextual fear conditioning test performance in 18–21-month-old mice**	Wheatley et al. (2019)
OGT ↑ in the liver	Tail vein injection of adenovirus Ad-*Ogt*	Insulin resistance	Yang et al. (2008)
		Advance the phase of *Bmal1* and *Clock* gene expression; ↑ the levels of *Per2*, *Cry1*, *Rorγ* and *Dbp* during the peak phase	Li et al. (2013)
OGT ↓ in the liver	Tail vein injection of Cre into *Ogt* floxed mice	Poorer glucose tolerance at ZT1, advanced the circulating glucose by 6–8 h, decreased *Bmal1* amplitude, and ↓ O-GlcNAcylation of BMAL1 and CLOCK	
**OGT ↓ in the liver**	**Tail vein injection of adenovirus Ad-*Ogt*-shRNA**	**Conversely, OGT knockdown in the liver using adenovirus with OGT shRNA decreased gluconeogenesis, ameliorated diabetes, and improved glucose homeostasis in db/db mice**	Ruan et al. (2012)
rAAV6-OGT; **rAAV6-OGA**	A single i.v. injection in mice 8 weeks after 55 mg/kg/d × 5 days at 6 weeks of age i.p. streptozotocin	rAAV6-OGT led to maladaptive cardiac remodeling and fibrosis; **rAAV6-OGA attenuated LV remodeling in diabetic mice**	Prakoso et al. (2018)

**FIGURE 4:**
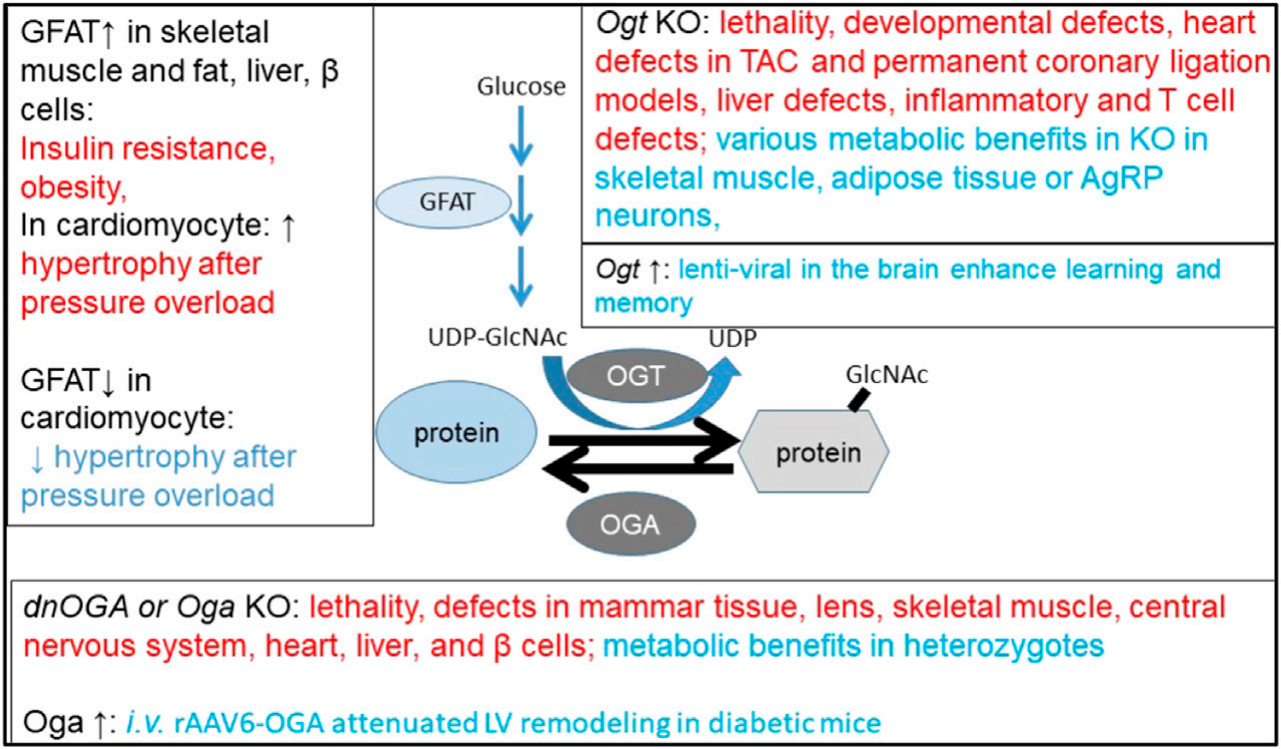
Beneficial and detrimental phenotypes of altering OGT, OGA, and GFAT. GFAT upregulation in skeletal muscle, fat, the liver, or β cells caused metabolic dysfunction and exacerbated hypertrophy after pressure overload in cardiomyocytes. GFAT knockout in cardiomyocyte attenuated hypertrophy after pressure overload. The cardiomyocyte phenotypes are associated with change of protein O-GlcNAcylation and mTOR activity. Ogt knockout or Oga knockout/dominant negative expression have both detrimental effects (mostly on tissue pathologies) and beneficial effects (on metabolism or learning and memory). Oga upregulation through AAV-i.v. injections seems to attenuate left ventricle remodeling in diabetic mice. Red highlights detrimental effects, and blue highlights beneficial effects.

Viral delivery of Cre or shRNA to induce downregulation of OGT or OGA and viral delivery to overexpress OGT or OGA through *i.v*. or *i.c.v.* helped to assess phenotypes of OGT or OGA deficiencies without developmental deficits or compensatory adaptations. So far, studies are still limited to collecting phenotypes and hypothesis generating identification of O-GlcNAc targets. What is still lacking is a firm handle on alteration of the O-GlcNAc proteome by these changes in different tissues and what changes are the most crucial for the various phenotypes and by what mechanisms functional pathways are impaired. A summary of the observed phenotypes is shown in Figure 4.

#### Using Pharmacological Reagents to Investigate the *In Vivo* Function of O-GlcNAcylation

Pharmacological reagents have been used to facilitate the investigations of the O-GlcNAc pathway function *in vivo* (Chatham et al., 2020). Here, we focus primarily on the studies using the best-characterized OGA inhibitor, thiamet G (Yuzwa et al., 2008) (Table 8). It has been shown that thiamet G has a K_i_ of ∼21 nM as assayed *in vitro* using recombinant human OGA and the pNP-GlcNAc substrate and an EC50 of ∼30 nM as determined by western blot densitometry analysis with CTD110.6 antibody in differentiated PC12 cells after exposure for 24 h (Yuzwa et al., 2008). The selectivity of thiamet G for OGA is >30,000-fold higher compared to other hexosaminidases, and the compound is stable in an aqueous solution with no sign of decomposition as assessed by ^1^H NMR after 9 days (Yuzwa et al., 2008). Thiamet G can cross the blood–brain barrier and be administered *i.v.* or through drinking water (Yuzwa et al., 2008; Yuzwa et al., 2012). Studies of the effects of thiamet G *in vivo* have largely focused on whether OGA inhibition modulates tau phosphorylation, as this is pertinent to whether OGA inhibition can be developed as a treatment against Alzheimer’s disease. We will a) summarize the studies using thiamet G to investigate the *in vivo* function of O-GlcNAcylation on tau phosphorylation and related neurological function, b) discuss studies using thiamet G to investigate *in vivo* functions of O-GlcNAcylation in other disease models, and c) discuss the development and studies of new OGA and potential OGT inhibitors.

**TABLE 8:** Investigating the effect of O-GlcNAcylation *in vivo* using thiamet G (22 references). Bold highlights indicate the beneficial effect. Those not highlighted indicate either the detrimental effect or that no biological or functional change was reported.

Amount used and duration	Observations	References
**Rat (6-week-old Sprague Dawley): *i.v*. 2, 10, or 50 mg/kg, 4 h**	**↑ protein O-GlcNAcylation at 10 and 50 mg/kg in total brain homogenate by western blot with CTD110.6, ↓ pS396 tau/Tau-5 (total)**	Yuzwa et al. (2008)
**Rat (6-week-old Sprague Dawley): *i.v.* 50 mg/kg, 1–16 h**	**↑ protein O-GlcNAcylation in total brain homogenate starting at 1 h and peak at 10 h, by western blot with CTD110.6; ↓ pS396 tau/Tau-5 starting at 1 h and peak at 4 h and restore to normal at 16 h**	
**Rat (21-week-old Long–Evans): drinking water, 200 mg/kg/d, 1 day**	**↑ protein O-GlcNAcylation in total brain homogenate by western blot with CTD110.6, ↓ pS396, pT231, and pS422/Tau-5, in total brain homogenate by western blot** **↓ pS396 immunostaining in CA1 and cortex; ↓ pS231 immunostaining in the cortex**	
Rat (6–8-week-old Sprague Dawley): i.p. 10 mg/kg	↑ protein O-GlcNAcylation in the hippocampal formation, CA1 pyramidal cells, inhibitory interneurons, and astrocytes 2, 8, or 24 h after injection, by CTD110.6 immunohistochemistry. No change of open-field behavior at 2 and 4 h after injection; no change of contextual fear conditioning 2 h after injection For novel object and placement recognition tests, exploration time at 2 h after injection was unchanged, while the performance in testing was significantly decreased in response to thiamet G	Taylor et al. (2014)
**Hemizygous female JNPL3 at 14 weeks of age: drinking water, 500 mg/kg/d, 1 week**	**↑ protein O-GlcNAcylation (using CTD110.6 antibody) in the brain; ↑ O-GlcNAcylation on Ser400 of tau/total tau (using an antibody generated against O-GlcNAc-Ser400/HT7 antibody) by western blot analyses**	Yuzwa et al. (2012)
**Hemizygous female JNPL3 mice at 9–12 weeks of age: drinking water, 500 mg/kg/d, ∼30 weeks**	**Alleviated body weight loss (administering 500 mg/kg/d thiamet G through drinking water for 22 weeks in wildtype mice did not change body weight, food or water consumption, organ weights, and motor neuron counts).** **With the differences in body weight, rotarod and cage hang tests were inconclusive**	
**Hemizygous female JNPL3 mice at 9–12 weeks of age: drinking water, 500 mg/kg/d, 36 weeks**	**Alleviated motor neuron loss. Immunohistochemistry studies demonstrated ↑ O-GlcNAcylation, ↓ pTau using AT8 antibody which detects S202 and T205 and S422 phosphorylation, ↓ S422 and Y29 sites of tau phosphorylation using pS422 and nY29 antibodies, and ↓ PHF-1 immunoreactivity**	
**RFP-GFP-LC3 mice at 4–8 weeks for 2 weeks: 500 mg/kg/d thiamet G through drinking water**	**↑ red puncta, unchanged yellow puncta, and ↓ p62 autophagic substrate were found in broad brain regions, indicating increased autophagic flux**	Zhu et al. (2018)
**JNPL3 hemizygous female mice at 9–12 weeks of age for 36 weeks: 500 mg/kg/d thiamet G through drinking water**	**↑ LC3 puncta, ↑ LC3II, ↓ P62, and unchanged p-MTOR/p-S6K and p-4EBP1 on western blots**	
**3×Tg-AD mice at 36–40 weeks for 2 weeks: 500 mg/kg/d thiamet G through drinking water**	**↓ LC3 puncta and p62 as measured by immunohistochemistry, LC3II was unchanged, and ↓ P62 by western blots**	
**Tau441 largest isoform wildtype human tau (175 µg/mouse, correlating to 700 µM in the brain) i.c.v. at 6 months**	**↑ protein O-GlcNAcylation in brain homogenates at 4.5–24 h after injection as demonstrated by western blot using RL2** **↓ tau phosphorylation at T181, T212, S214, S262/S356 S404, and S409, ↑ Tau phosphorylation at S199, S202, S396, and S422, and unchanged at T217 by western blot using site-specific antibodies** **↓ pS473-AKT, total GSKβ, and pS9-GSK3β were also evident by western blot**	Yu et al. (2012b)
Female Thy-1-tau P301L, 4–8 h thiamet G up to 500 mg/kg	↑ protein O-GlcNAcylation in brain homogenates by western blot using CTD110.6 No change of total or p-tau (Tau5, pS396, pS404, AD2, AT180, or 22E8)	Borghgraef et al. (2013)
**Female Thy-1-tau P301L, 3 days drinking water at 2.5 mg/ml**	**Ameliorated upper-airway breathing defects of these mice**	
**Female Thy-1-tau P301L, 2.5 months drinking water at 2.5 mg/ml**	**Improved clasping score, body weight, and morbidity**	
**Female double-transgenic APPSwe-Tau mice at 10–23 weeks of age drinking water at 200 or 500 mg/kg/d based on ∼4 ml/d/animal for 34 weeks**	**After 20 weeks, the 500 mg/kg/d group exhibited better performance in a Morris water maze probe trial. After 34 weeks, ↑ global protein O-GlcNAcylation in brain homogenates by western blot using either RL2 or CTD110.6, in both 200 and 500 mg/kg/d groups, ↑ immunohistochemical signals in the 500 mg/kg/d group in the hippocampus, cerebellum, pons, and amygdala using RL2 and CTD110.6; total tau or p-tau were unchanged (92e, pS199/pS202 pT205, pT212, pT214, pT217 pT231, pS262/pS356 (12E8), and pS396/pS404 (PHF-1) for western blot; pS396, pS396/pS404 (PHF-1), pT231 (AT180), and pS262/pS356 (12E8) for immunostaining)** **↓ Aβ42 in the 500 mg/kg/d group using ELISA assay** **↓ plaques in in the cortical and hippocampal regions in the 500 mg/kg/d group and decreased plaque in the cortex in the 200 mg/kg/d group using immunohistochemistry with the 6E10 antibody**	Yuzwa et al. (2014)
**rTg(tauP301L)4510 mice with acute (1day), subchronic (14days), and chronic (4 months) thiamet G in water administered p.o. at 500 mg/kg/d beginning at 2 months of age**	**↑ protein O-GlcNAcylation in brain homogenates both at 1 day and 14 days as measured by RL2 western blots** **O-tau at S400 was ↑ at 14 days but not 1 d. pS202, pS396, pS356, and pS262 were ↓ at 1 day but not 14 d. pS400 was unchanged** **Chronic thiamet G ↓ pS202, pS396, pS356, pS262, and HT7 tau at 64 kD after high-speed spin (the pellet of 110,000 g × 15 min spin) but not at 50–60 kD (supernatant of the spin)** **Chronic thiamet G ↓ pS202/205 and AT8 tau immunoreactivity**	Graham et al. (2014)
Thiamet G at 10 and 500 mg/kg a single oral dose of water, 6 h	The brain-to-plasma ratio of thiamet G was shown to be < 0.1 as measured by LC-MS, ↑ 1.7× and 4 × of protein O-GlcNAcylation as measured by the quantitative sandwich immunoassay with both wheat germ agglutinin and the RL2 antibody	Hastings et al. (2017)
**rTg4510 mice fed thiamet G at either 8 weeks or 12 weeks of age for 8 or 4 weeks duration at 3.3 mg/g in chow, achieving ∼500 mg/kg/d**	**In the 8-week but not the 4-week duration group:** **↓ PHF6, p-Thr Tau, and Tau aggregation without changing total Tau, as assessed by an AlphaLISA-based immunoassays in the brain insoluble fractions. ↓ CSF total Tau** **↑ PNGase-resistant and β-elimination-sensitive O-tau, as assessed by click chemistry, but not RL2 or immunoprecipitation of anti-O-tau antibody 3,925**	Hastings et al. (2017)
**Young mice i.p. 30 mg/kg 18 h before tMCAO** **Young or old mice i.p. 30 mg/kg 30 min after onset of pMCAO**	**↓ infarct**	Jiang et al. (2017)
**Young mice i.p. 20 mg/kg/d for 3 days before tMCAO or 30 min after onset of tMCAO daily till day 3**	**Neurobehavioral performance was improved** **↓ infarct** **↑ IL10, ↓ IL6, G-CSF, TNFα, and IL1β in both groups** **↓ microglia activation as measured by Iba1+, CD16/32+, Cox-2+, iNOS+ cells**	He et al. (2017)
**In rat spinal cord injury (SCI) model, i.v. injected within 1 h after injury at 10 mg/kg/d x 3 days**	**↓ Cleaved caspase 3 at 24 h after SCI, ↓ CD68^+^ microglia at 7days after SCI improved neurological score at 5–20 days following SCI** **↓ histological alterations of the injured spinal cord at 24 h and 21 days after SCI**	Liang et al. (2019)
**Rats that have been injected kainate to induce seizure**	**Progressively↓ seizure severity after consecutive daily thiamet G injection (10 mg/kg i.p.)**	Sanchez et al. (2019)
Single *i.p.* injection at 0, 10, 20, 100, 200, or 500 mg/kg	↑ protein O-GlcNAcylation at all these doses and peaked at 20 mg/kg, 8 h after injection in the brain, liver, and knee using western blot with RL2; no change in the muscle at any of these doses	Andres-Bergos et al. (2012)
Male C57BL/6 mice at 23 days i.p. 20 mg/kg/d × 15 days	↑ protein O-GlcNAcylation in the brain, liver, and muscle ↑ growth plate height and hypertrophic zone height in the endochondral plate of the tibias, suggesting a stimulation of growth plate chondrocyte differentiation	
In a mouse tibialis anterior muscle injury model: i.p. 40 mg/kg 1 day after injury for 3 days	↓ myogenin levels in tibialis anterior muscle	Kim et al., (2020)
STZ was injected *i.p.* at 50 mg/kg for 5 days, thiamet G was injected *i.v.* 20 mg/kg/wk 1 week after STZ for 8 weeks	↑ vascular calcification in STZ-treated mice	Heath et al. (2014)
C57BL/6 mice at 8 weeks of age: *i.p.* 20 mg/kg/d × 15 days	↑ protein O-GlcNAcylation in the colon as assessed by western blots using RL2 The increase of protein O-GlcNAcylation *i.p.* injected with thiamet G (20 mg/kg/d) for 15 days ↑ OGA and ↓ OGT at both protein and mRNA level in the colon	Olivier-Van Stichelen et al., (2014), Decorcelle et al. (2020)
Single oral administration of MK-8719 to SD rats	↑ Brain protein O-GlcNAcylation at 0.3 mg/kg; ↑ PBMC protein O-GlcNAcylation at 10 mg/kg, using a sandwich immunoassay with wheat germ agglutinin and RL2 ↑ protein O-GlcNAcylation at 30 mg/kg, 8–24 h in both brain and PBMC, with brain greater ↑ at 12 h MK-8719 levels peaked at 2 h in both the brain and plasma	Wang et al. (2020)
rTg4510 mice at 8 weeks of age: Chronic diet dosing of MK-8719 at 10, 30, and 100 mg/kg	No overt adverse effects from 8 to 16 weeks of age **↓ aggregated tau after 30 mg/kg, ↓ AT8 tau after 10 mg/kg, and ↓ PHF6 tau after 30 mg/kg in insoluble tau fraction using AlphaLISA-based immunoassays**	
rTg4510 mice at 8 weeks of age: chronic diet dosing of MK-8719 at 100 mg/kg	**↓ brain NFT pathology using AT8 immunostaining; ↓ CSF total tau after 100 mg/kg from 8 to 20 weeks** ↓ spontaneous locomotor activity in terms of distance traveled 8 weeks after of MK-8719 in these mice compared to age-matched mice with vehicle **↑ cortex and hippocampal volume were also seen 16 and 24 weeks after MK-8719**	

##### Using Thiamet G to Investigate the *in vivo* Function of O-GlcNAcylation on Tau Phosphorylation and Related Neurological Function

After thiamet G synthesis and characterization, the first set of studies has performed *i.v.* injection of 6-week-old male Sprague Dawley rats, and drinking water administration of 21-week-old male Long–Evans rats. Dose response characterization by western blot in total brain homogenates with CTD110.6, pS396, and Tau-5 antibodies has demonstrated increased global protein O-GlcNAcylation and decreased pS396 tau/Tau (total) after *i.v.* injection in rats at 10 or 50 mg/kg dose but not 2 mg/kg for 4 h (Yuzwa et al., 2008). Time course studies by western blot with the CTD110.6 antibody demonstrated that *i.v.* injection in rat at 50 mg/kg increased global protein O-GlcNAcylation in total brain homogenates, starting at 1 h and peaking at 10 h and decreased pS396 tau/Tau-5 starting at 1 h, peaking at 4 h, and restoring to normal at 16 h (Yuzwa et al., 2008). Administering thiamet G in the drinking water of rats at 200 mg/kg/d for 1 day increased global protein O-GlcNAcylation in total brain homogenates as assessed by western blot analyses with the CTD110.6 antibody and decreased pS396, pT231, and pS422/Tau-5 in total brain homogenates as assessed by western blot analyses using respective antibodies (Yuzwa et al., 2008). Furthermore, thiamet G in drinking water at 200 mg/kg/d for 1 day also decreased pS396 immunostaining in both hippocampal CA1 and cortex and pS231 immunostaining in the cortex (Yuzwa et al., 2008). These experiments suggested that thiamet G delivery might be a strategy to alleviate p-tau-associated proteotoxicity in the brain and to be considered as a treatment in Alzheimer’s disease.

Following the initial study, other routes of administration, time courses, and concentrations have been tested for functional relevance in the brain. Sprague Dawley male rats at 6–8 weeks of age were then *i.p.* injected with thiamet G at 10 mg/kg and analyzed 2, 8, or 24 h after injection. Immunohistochemistry using the CTD110.6 antibody has demonstrated increased protein O-GlcNAcylation in the hippocampal formation, including in the CA1 pyramidal cells, inhibitory interneurons, and astrocytes. No change of open-field behavior at 2 and 4 h after injection and no change of contextual fear conditioning 2 h after injection were observed. However, for novel object and placement recognition tests, the exploration time for the control and thiamet G group at 2 h after injection was similar, while the novel object recognition and novel placement recognition performance in the testing period were significantly decreased in response to thiamet G. This study suggests that thiamet G injection may have a significantly detrimental effect on hippocampal-dependent memory in normal animals (Taylor et al., 2014). In contrast to the initial study, which focused on p-tau, this study used a different injection route and examined the behavioral changes after thiamet G (Taylor et al., 2014).

Mouse studies have used higher thiamet G doses and treatment duration in hemizygous female JNPL3 mice overexpressing mutant human P301L tau under the mouse prion promoter (Yuzwa et al., 2012). Mice 14 weeks of age consuming 500 mg/kg/d thiamet G in drinking water once per week showed increased global protein O-GlcNAcylation (using CTD110.6 antibody) in the brain, as well as O-GlcNAcylation on S400-tau/total tau (using an antibody generated against O-GlcNAc-S400/HT7 antibody) (Yuzwa et al., 2012). Administration of 500 mg/kg/d thiamet G through drinking water at 9–12 weeks of age for 30–36 weeks in JNPL3 mice alleviated body weight and motor neuron loss. With the differences in body weight, rotarod and cage hang tests were inconclusive. A control experiment administering 500 mg/kg/d thiamet G through drinking water for 22 weeks in wildtype mice did not change body weight, food or water consumption, organ weights, and motor neuron counts. Immunohistochemistry studies demonstrated increased O-GlcNAcylation, decreased p-tau using AT8 antibody which detects S202 and T205 phosphorylation, decreased p-tau using pS422 and nY29 antibodies, and decreased PHF-1 immunoreactivity which correlates with pathological tangles (Yuzwa et al., 2012). These two initial studies suggest that, in JNPL3 mice, inhibition of OGA by thiamet G significantly decreased p-tau and may be beneficial in attenuating p-tau-dependent pathology.

Regarding potential effects of O-GlcNAcylation on protein turnover by autophagy, using genetic models and site-directed mutagenesis, one study reported that increased O-GlcNAcylation of SNAP29 inhibits autophagy in a variety of cell cultures (Guo et al., 2014), and another study reported that increased O-GlcNAcylation of GRASP55 also inhibits autophagy (Zhang et al., 2018). Both studies indicated inhibition at later stages of autophagy, likely due to attenuated autophagosome-lysosome fusion events. With regard to neurodegenerative diseases, one study indicated that, in primary neuron cultures, thiamet G attenuated autophagic flux, which involves mTOR phosphorylation (Wani et al., 2017c), although a different report found that thiamet G increased autophagy in an mTOR-independent manner (Zhu et al., 2018). The differences between these two reports may be related to the different culture conditions (whether neurons were cocultured with astrocytes), the amount of thiamet G used, and/or thiamet G purity. The latter study also investigated the effect of thiamet G on autophagy *in vivo*. To do this, thiamet G was administered through drinking water at 3.75 mg/ml to RFP-GFP-LC3 mice starting at 4–8 weeks of age. Based on an average animal weight of 30 g and water consumption of 4 ml/d/mouse, this was estimated to be equivalent to 500 mg/kg/d. After 2 weeks, increased red puncta, unchanged yellow puncta, and decreased p62 autophagic substrate were observed in broad brain regions, indicating increased autophagic flux. Increased LC3 puncta, as well as increased LC3II, decreased P62, and unchanged p-MTOR/p-S6K and p-4EBP1 on western blots, were reported for JNPL3 hemizygous female mice dosed with thiamet G starting at 9–12 weeks of age for 36 weeks. When 3xTg-AD mice were dosed at 36–40 weeks for 2 weeks, LC3 puncta and p62 were decreased as measured by immunohistochemistry, LC3II was unchanged, and p62 was decreased by western blots (Zhu et al., 2018). It is likely that the effect of thiamet G on autophagy depends on the amount, route, timing, and duration of delivery, the specific cell types, and the age and sex of the animals.

Additional studies have explored the impact of thiamet G on other tau- or Aβ-based mouse models of Alzheimer’s disease. With these additional studies, different routes, doses, and durations of thiamet G administration have been evaluated. To minimize systemic influence, thiamet G (175 µg/mouse, correlating to 700 µM in the brain) has been injected into the lateral ventricle of tau transgenic mice overexpressing the largest isoform of wildtype human tau under the PDGF promoter, at 6 months of age (Yu et al., 2012b). Global protein O-GlcNAcylation in brain homogenates was elevated at 4.5–24 h after injection as demonstrated by western blot analysis using the RL2 antibody (Yu et al., 2012b). Significant decreases of pS473-AKT, total GSKβ, and pS9-GSK3β were observed (Yu et al., 2012b). Tau phosphorylation was decreased at T181, T212, S214, S262/S356 S404, and S409, increased at S199, S202, S396, and S422, and unchanged at T217, as shown by western blot using site-specific antibodies (Yu et al., 2012b). These studies suggest that specific residues in tau may be affected by thiamet G differently. The different responses of these amino-acid residues to thiamet G may be due to secondary effects of changed kinase and phosphatase activities which themselves may be O-GlcNAcylated and changed tau conformation resulting in specific amino acids being more or less accessible to OGT vs. OGA. Another possibility is a change OGT/OGA activities in different cellular compartments due to cross regulation.

In addition to the abovementioned *i.v., i.p., i.c.v*., or drinking water administration methods, thiamet G can also be administered by gavage. One study used female transgenic mice expressing the longest human tau isoform with the P301L mutation under the mouse Thy-1 promoter. This study reported that 4–8 h of thiamet G (up to 500 mg/kg) *i.p.,* gavage, or through drinking water did not decrease total or p-tau (Tau5, pS396, pS404, AD2, AT180, or 22E8) despite significant increase of global protein O-GlcNAcylation as demonstrated by western blot using CTD110.6 (Borghgraef et al., 2013). Thiamet G in drinking water at 2.5 mg/ml for 3 days, however, ameliorated upper-airway breathing defects of these mice and, for a duration of 2.5 months, improved the clasping score, body weight, and morbidity (Borghgraef et al., 2013). These studies suggest that thiamet G may impact phenotypes due to P301L overexpression in a p-tau-independent manner.

Thiamet G was also administered through drinking water at 200 or 500 mg/kg/d based on ∼4 ml/d/animal for 34 weeks, to female double-transgenic APPSwe-Tau mice at 10–23 weeks of age (Yuzwa et al., 2014). After 20 weeks of administration, the 500 mg/kg/d group exhibited better performance in the Morris water maze probe trial. At the end of the study, global protein O-GlcNAcylation in brain homogenates was increased as assessed by western blot using either RL2 or CTD110.6. Immunohistochemical signals were increased in the 500 mg/kg/d group in the hippocampus, cerebellum, pons, and amygdala (Yuzwa et al., 2014). Total tau or p-tau was unchanged (92e, pS199/pS202, pT205, pT212, pT214, pT217, pT231, pS262/pS356 (12E8), and pS396/pS404 (PHF-1) by western blot; pS396, pS396/pS404 (PHF-1), pT231 (AT180), and pS262/pS356 (12E8) by immunostaining) (Yuzwa et al., 2014). Using ELISA, it was shown that Aβ42 was decreased in the 500 mg/kg/d group (Yuzwa et al., 2014). Immunohistochemistry studies using 6E10 antibody detected decreased plaque in the cortical and hippocampal regions in the 500 mg/kg/d group and decreased plaque in the cortex in the 200 mg/kg/d group (Yuzwa et al., 2014). These studies suggest that thiamet G may impact cognition in a p-tau-independent manner.

A time-course study used rTg(tauP301L)4510 mice with acute (1 day), subchronic (14 days), and chronic (4 months) thiamet G in water administered *p.o.* at 500 mg/kg/d beginning at 2 months of age (Graham et al., 2014). It was found that at both 1 day and 14 days after administration, protein O-GlcNAcylation in brain homogenates as measured by RL2 western blots was changed. O-tau at S400 was increased at 14 days but not 1 d. pS202, pS396, pS356, and pS262 were decreased at 1 day but not 14 days. pS400 was unchanged. Chronic thiamet G decreased pS202, pS396, pS356, pS262, and HT7 tau at 64 kD after a high-speed spin (the pellet of 110,000 g × 15 min spin) but not at 50–60 kD (supernatant of the spin) (Graham et al., 2014). pS202/205 and AT8 tau immunoreactivity was decreased in the hippocampus (Graham et al., 2014).

Another study measured brain and plasma thiamet G levels after oral administration. This is important, as the ability of thiamet G to cross the blood brain barrier is essential if one wants to ensure its effect in the central nervous system with a systemic delivery, without risk of systemic side effects. In this study, the brain to plasma ratio of thiamet G was shown to be < 0.1 as measured by LC-MS after thiamet G at either 8 weeks or 12 weeks of age for eight or 4 weeks of duration at ∼500 mg/kg/d (Hastings et al., 2017). In this study, administration of thiamet G at 10 and 500 (3.3 mg/g in chow) mg/kg led to a 1.7- and 4-fold increase of protein O-GlcNAcylation in brain homogenates using a quantitative sandwich immunoassay with wheat germ agglutinin and the RL2 antibody (Hastings et al., 2017). The rTg4510 mice in the FVB/129S6 background were also fed with thiamet G at either 8 weeks or 12 weeks of age for eight or 4 weeks of duration at ∼500 mg/kg/d (Hastings et al., 2017). The group of mice fed with thiamet G for 8 weeks exhibited decreased PHF6, p-Thr Tau, and Tau aggregation, without changing total Tau, as assessed by AlphaLISA-based immunoassays in the brain insoluble fractions. This 8-week thiamet G duration also decreased CSF total Tau. Using click chemistry, but not RL2 or anti-O-tau antibody, 3,925 immunoprecipitation, PNGase-resistant, and β-elimination-sensitive O-tau were detected and found to be increased by 8 weeks of thiamet G (Hastings et al., 2017).

##### Using Thiamet G to Investigate the *In Vivo* Function of O-GlcNAcylation on Functions Not Directly Related to Tau

Although the study of OGA inhibitors *in vivo* started at decreasing p-tau and most work focused on effects on Alzheimer’s disease animal models, thiamet G has also been used in other disease models. One such study used *Xbp1*f/f;Emx1-Cre (Xbp1-cKO), *Xbp1*f/f (control), and CaMK2a-tTA;TRE-XBP1s transgenic mice to investigate the role of *Xbp1* (X-box binding protein-1) in a stroke model with transient middle cerebral artery occlusion (tMCAO) and permanent MCAO (pMCAO). It was found that XBP1 overexpression increases GFAT1 protein in the cortex and protein O-GlcNAcylation in the cortex and hippocampus as measured by western blot using CTD110.6 antibody. Protein O-GlcNAcylation was increased in the ipsilateral side 3 h after tMCAO. XBP1 cKO attenuated this increase after tMCAO and worsened infarct 24 h after tMCAO and on day 3 after pMCAO. In contrast to young mice (2–3 months), aged mice (22–24 months) did not exhibit increased protein O-GlcNAcylation 6 h after pMCAO. *i.p.* injection with 30 mg/kg thiamet G 18 h before tMCAO or 30 min after onset of pMCAO both decreased infarct area in young mice. In comparison, *i.p.* injection with 30 mg/kg thiamet G 30 min after the onset of pMCAO both decreased infarct and improved neurological scores on day 3 in aged mice (Jiang et al., 2017).

In another study of tMCAO, young mice (10–12 weeks) were used. Thiamet G was *i.p.* injected at 20 mg/kg/d for 3 days before MCAO or 0.5 h after MCAO daily until day 3. Neurobehavioral performance was improved, infarct decreased, IL10 increased, and IL6, G-CSF, TNFα, and IL1β decreased in both pretreatment and posttreatment groups. Both thiamet G groups also exhibited decreased microglia activation as measured by staining Iba1^+^, CD16/32^+^, Cox-2^+^, and iNOS^+^ cells (He et al., 2017). In the rat spinal cord injury (SCI) model, thiamet G was *i.v.* injected within 1 h after injury at 10 mg/kg/d for 3 days. This dosing decreased cleaved caspase 3 at 24 h after SCI, decreased CD68 ^+^ microglia at 7 days after SCI, improved neurological score at 5–20 days following SCI, and mitigated histological alterations of the injured spinal cord at 24 h and 21 days after SCI (Liang et al., 2019). In a rat kainate acid-induced seizure model, 3 consecutive daily thiamet G injections (10 mg/kg i.p.) progressively decreased seizure severity (Sanchez et al., 2019). Since it was found that hippocampal OGT is decreased in epileptic rats, presumably, some of the key proteins had decreased O-GlcNAcylation due to OGT downregulation. Thiamet G may be able to elevate O-GlcNAcylation of these key proteins, which may play an important role for synaptic activities that are inhibitory of epileptic activities. This study tested two of these candidate proteins, sortilin-related receptor (SORL1) and tropomodulin 2 (Tmod2), which showed decreased O-GlcNAcylation in epileptic rats in a proteomics study, yet found no significant increase in O-GlcNAcylation after thiamet G by immunoprecipitation and western blot. In human TLE resected tissue, thiamet G can increase both OGT and OGA protein levels. It is also important to note that although 3 consecutive daily thiamet G injections attenuated seizure severity, chronic inhibition of OGA (10 mg/kg/d × 2 weeks) resulted in hippocampal atrophy as assessed by MRI (Sanchez et al., 2019).

Thiamet G effects have also been examined in tissues other than the brain. In a chondrocyte hypertrophy study, single *i.p.* injection at 0, 10, 20, 100, 200, or 500 mg/kg was performed with one mouse each. Western blot with the RL2 antibody showed that 8 h after injection, protein O-GlcNAcylation was increased, by all of the doses, and peaked at 20 mg/kg in the brain, liver, and knee, except in the muscle where no changes were observed. In 23-day-old male C57BL/6 mice *i.p.* injected 20 mg/kg/d for 15 days, protein O-GlcNAcylation was elevated in the brain, liver, and muscle. Growth plate height and hypertrophic zone height in the endochondral plate of the tibias were increased by thiamet G, suggesting a stimulation of growth plate chondrocyte differentiation (Andres-Bergos et al., 2012).

In a mouse skeletal muscle injury model (in the tibialis anterior muscle), thiamet G (40 mg/kg) was injected *i.p.* 1 day after injury for 3 days. It was shown that myogenin levels in tibialis anterior muscle were decreased in the thiamet G group at 4 days after injury, suggesting an impact on myoblast differentiation (Kim et al., 2020). In a streptozotocin- (STZ-) induced diabetes model, STZ was injected *i.p.* 50 mg/kg/d for 5 days and thiamet G was injected 1 week after STZ *i.v.* 20 mg/kg/wk for 8 weeks. In this study, thiamet G further increased vascular calcification in STZ-treated mice (Heath et al., 2014). C57BL/6 mice at 8 weeks of age were injected with thiamet G *i.p.* 20 mg/kg/d for 15 days and fasted for 24 h before harvesting, and protein O-GlcNAcylation was found increased in the colon by western blot using RL2 (Olivier-Van Stichelen et al., 2014). The increase of protein O-GlcNAcylation in mice *i.p.* injected with thiamet G (20 mg/kg/d) for 15 days has also been shown to lead to concomitant increase of OGA and decrease of OGT at both protein and mRNA levels in the colon (Decourcelle et al., 2020).

**FIGURE 5:**
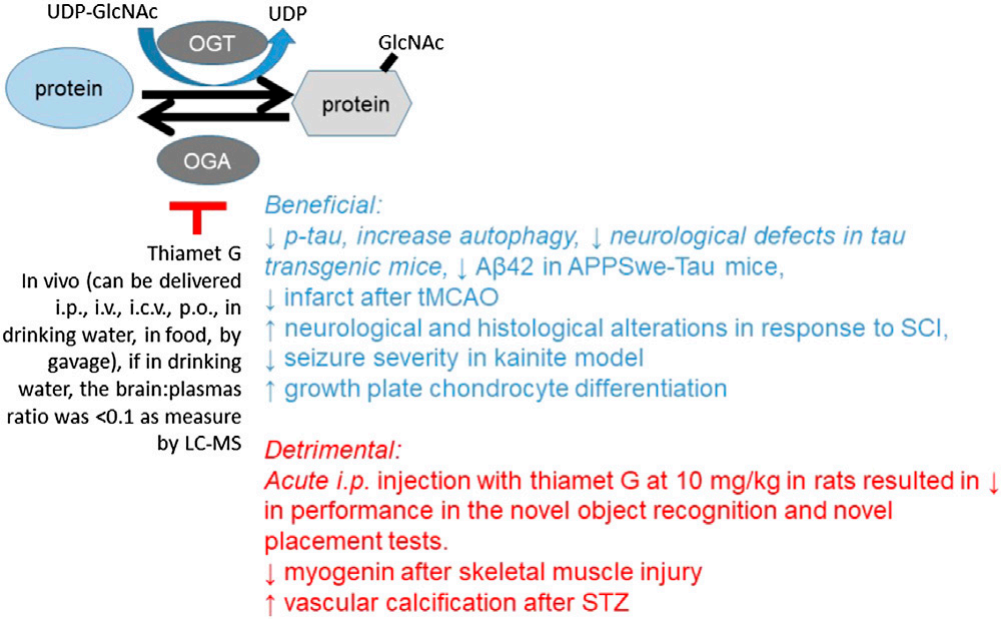
Beneficial and detrimental effects of thiamet G. Thiamet G can be delivered via many routes and is shown to increase protein O-GlcNAcylation by using an antibody-based method. In multiple transgenic tau or APP/tau models, thiamet G has been shown to be beneficial in decreasing p-tau or Aβ42 (shown in blue). However, there are a few studies which demonstrated that thiamet G can be detrimental in decreasing learning and memory and in increasing damage after skeletal muscle or pancreatic β cells (shown in red).

Thiamet G has been delivered to rodents *in vivo* through *i.p., i.v., i.c.v., p.o.,* drinking water, food, or by gavage. All methods have been shown to increase overall protein O-GlcNAcylation by antibody-based detection if thiamet G was in sufficient amounts and within a broad range of acute, sub-acute or chronic durations. The penetration of thiamet G through the blood brain barrier is evident albeit with low efficiency. One study demonstrated that when thiamet G was delivered through drinking water, the brain:plasma ratio was <0.1 as measured by LC-MS. With these methods of delivery, current studies have found both beneficial and detrimental effects of thiamet G in various pathological models. With regard to the brain, in most studies using tau transgenic or APPSwe/tau models, thiamet G is beneficial in decreasing p-tau and Aβ42, except in one study, which reported decreased learning and memory in novel object/placement recognition tests. There are studies demonstrating beneficial effects of thiamet G in tMCAO and seizure models and growth plate chondrocyte differentiation. However, there are also detrimental effects in skeletal muscle or pancreatic β-cell injury models. These studies are summarized in Figure 5.

##### The Development and Validation of New OGA and OGT Inhibitors

**FIGURE 6:**
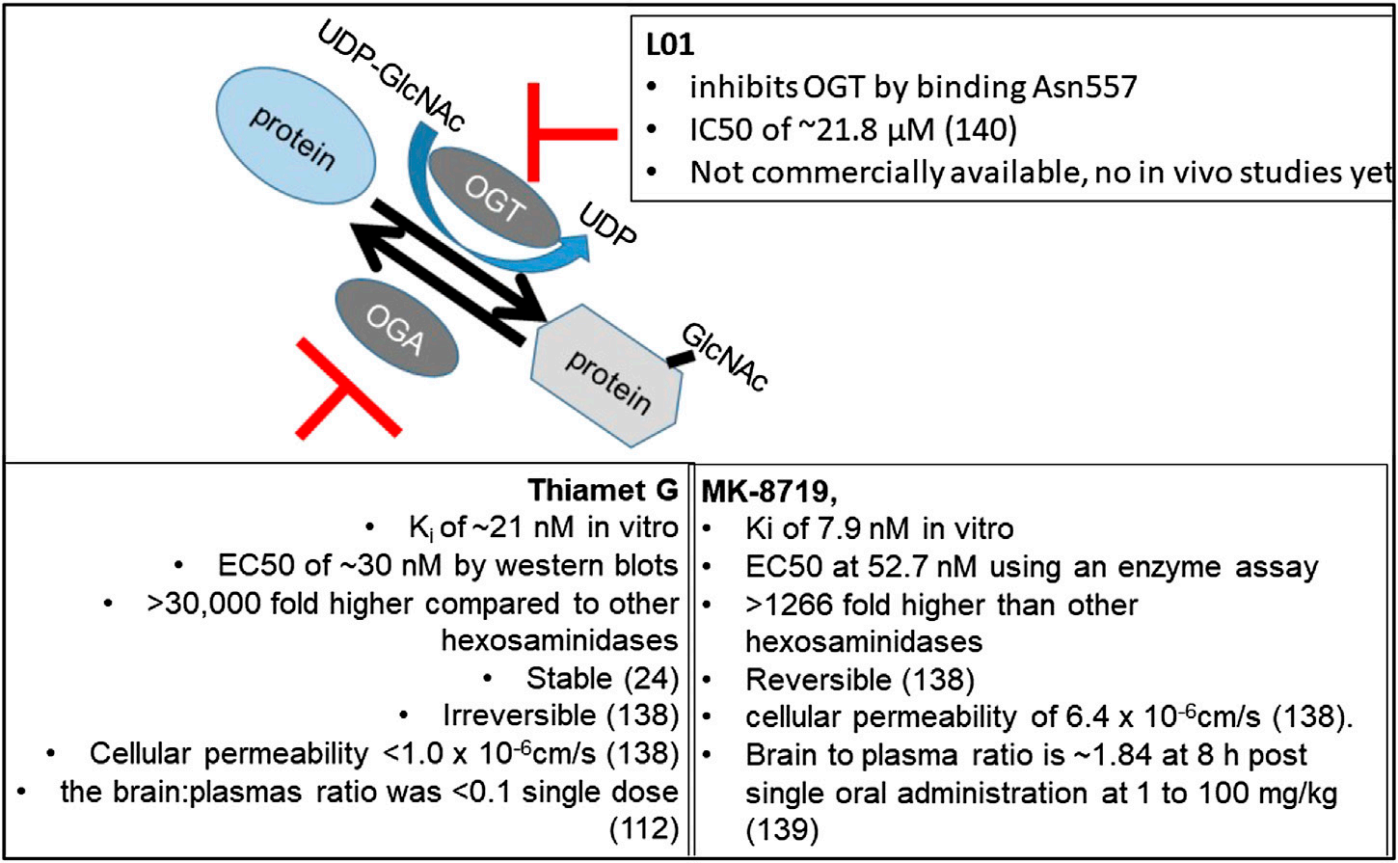
Thiamet G vs. MK-8719 and L01. OGA inhibitors (thiamet G and MK-8719) and OGT inhibitor L01 are compared here. Both thiamet G and MK-8917 have low Ki and EC50, and both are highly selective for OGA; MK has a higher cellular permeability and brain-to-plasma ratio, although it is not yet commercially available, and thus, fewer studies have used MK-8917. OGT inhibitors are still with Ki/IC50 of µM range and not commercially available, and no *in vivo* studies have been reported yet.

Although the impact of thiamet G on brain protein O-GlcNAcylation has been demonstrated, its brain penetration is low (Hastings et al., 2017). To improve the translational potential of using OGA inhibitors as therapeutic agents, a novel OGA inhibitor MK-8719 has been developed. MK-8719 has a Ki of 7.9 nM against purified human OGA, with EC50 for PC-12 cells at 52.7 nM using an OGA enzyme assay, and Ki to human lysosomal β-hexosaminidase >10,000 nM (Selnick et al., 2019). These properties are similar to the reported properties of thiamet G (Selnick et al., 2019). In contrast to thiamet G, however, MK-8719 binding to OGA was reversible with a dissociation half-life of 46 s and had a higher cellular permeability of 6.4 × 10^−6 ^cm/s (Selnick et al., 2019). The brain:plasma ratio was also higher at ∼1.84 at 8 h after single oral administration at 1–100 mg/kg (Wang et al., 2020) (Figure 6).

Dosing with a single oral administration of MK-8719 to Sprague Dawley (SD) rats at 0.3 mg/kg increased brain protein O-GlcNAcylation and at 10 mg/kg increased PBMC protein O-GlcNAcylation, using a sandwich immunoassay with wheat germ agglutinin and RL2. At 30 mg/kg, 8–24 h, both brain and PBMC exhibited increased protein O-GlcNAcylation with brain exhibiting a greater increase at 12 h, while MK-8719 levels peaked at 2 h (Wang et al., 2020). An oral single dose of a brain-penetrable PET tracer [^18^F]MK-8553 enabled PET scans 4 h after dosing in SD rats, rTg4510 mice, and monkeys and revealed a similar distribution in these animals, with the highest uptake in the striatum and lower in the cerebellum (Wang et al., 2020). Chronic diet dosing at 10, 30, and 100 mg/kg from 8 to 16 weeks of age in rTg4510 mice found no overt adverse effects. Using AlphaLISA-based immunoassays, it was shown that the insoluble tau fraction had decreased aggregated tau and PHF6 tau with 30 mg/kg and decreased AT8 tau with 10 mg/kg. Brain NFT pathology assessed by AT8 immunostaining and CSF total tau were found decreased after 100 mg/kg from 8 to 20 weeks. Spontaneous locomotor activity in terms of distance traveled was decreased 8 weeks after MK-8719 in these mice compared to age-matched rTg4510 mice with vehicle (Wang et al., 2020). Increased cortex and hippocampus volumes were also seen 16 and 24 weeks after MK-8719 (Wang et al., 2020) (Table 8).

OGT inhibitors have also been investigated. Through structure-based virtual screening analysis, L01 has been found to interact with Asn557 in OGT and inhibits its activity with IC50 of ∼21.8 µM (Liu et al., 2017). Recent studies also started to develop high-affinity OGT inhibitors (Kd nM range), such as OSMI-1 and OSMI-4 (Martin et al., 2018). Subsequently, a compound ES1 has been designed and synthesized to react with C917 to inactivate OGT and at µM levels can decrease global protein O-GlcNAcylation (Worth et al., 2019). So far, these inhibitors are not commercially available and have not been reported to be effective *in vivo* in rodent models. So, the search continues.

## Conclusions and Future Directions

**FIGURE 7:**
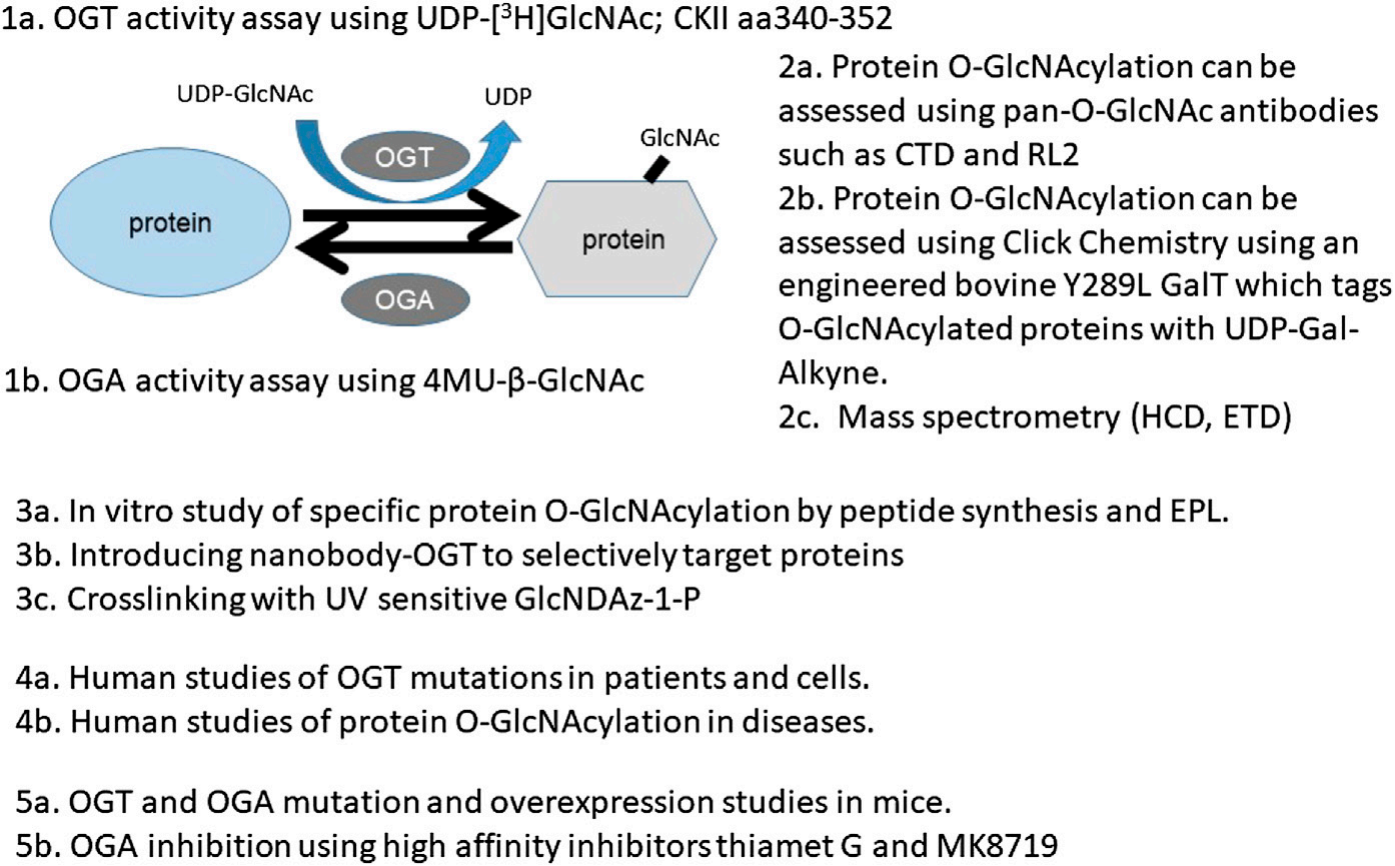
Approaches in studying protein O-GlcNAcylation. This review highlights some of the key approaches in studying protein O-GlcNAcylation and major findings in human and mice. This include 1) techniques in assessing OGT and OGA enzymatic activity using purified proteins or cell/tissue extracts; 2) techniques in assessing global and specific protein O-GlcNAcylation using antibodies, click chemistry, and mass spectrometry; 3) techniques in studying specific protein O-GlcNAcylation *in vitro* and selectively investigating specific protein O-GlcNAcylation *in vivo*; 4) the observations of consequences of OGT mutation in humans and association of changes of global protein O-GlcNAcylation in specimens from human diseases; and 5) investigations of protein O-GlcNAcylation in mice using OGT/OGA transgenic mouse models or pharmacological inhibitors of OGA.

O-GlcNAcylation is a posttranslational modification discovered in the 1980s, while quickly gaining attention due to its sensitivity to nutrients and stress and its potential to interfere with other protein modifications, especially phosphorylation at Ser/Thr residues. Over the years, biochemical, pharmacological, and genetic tools have been developed to facilitate the investigation of its biological function. We discuss here currently accessible and advanced tools, as well as observations in studying the regulation and functional consequence of protein O-GlcNAcylation *in vivo* (Figure 7). Technical and conceptual gaps in understanding O-GlcNAc biology include, but are not limited to, the following:

- Lack of effective tools to quantify precise protein O-GlcNAcylation levels down to the individual amino-acid residues *in vivo* without introducing tags and mutations;
- Lack of tools to quantify the intracellular localization of O-GlcNAcylated proteins *in vivo* without introducing tags and mutations;
- Lack of integrated studies of how O-GlcNAcomes are regulated and how O-GlcNAcylation enzyme activities affect transcriptomes, proteomes, and metabolomes in both healthy and pathological states.

Research efforts toward these directions will be crucial to provide insights into O-GlcNAc biology, how it integrates and signals cellular function and organismal physiology, and how its perturbation affects disease progression.
